# Transcriptomic Profiling Reveals Differences in Slow‐Twitch and Fast‐Twitch Muscles of a Cigarette Smoke‐Exposed Rat Model

**DOI:** 10.1002/jcsm.13633

**Published:** 2024-11-29

**Authors:** Timothy K. H. Fung, Kwok Kuen Cheung, Xia Wang, Benson W. M. Lau, Shirley P. C. Ngai

**Affiliations:** ^1^ Department of Rehabilitation Sciences The Hong Kong Polytechnic University Kowloon Hong Kong; ^2^ School of Medicine The Chinese University of Hong Kong Shenzhen China

**Keywords:** chronic obstructive pulmonary disease, cigarette smoke, fibre type shifting, muscle degradation, rodents, skeletal muscle

## Abstract

**Background:**

Cigarette smoking is known to affect muscle function and exercise capacity, including muscle fatigue resistance. Most studies showed diminished cross‐sectional area and fibre type shifting in slow‐twitch muscles such as the soleus, while effects on fast‐twitch muscles were seldom reported and the differential responses between muscle types in response to exposure to cigarette smoke (CS) were largely unknown. This study aimed to elucidate the histomorphological, biochemical and transcriptomic changes induced by CS on both slow‐twitch and fast‐twitch muscles.

**Method:**

Male Sprague–Dawley rats were randomly divided into two groups: sham air (SA) and CS. The rats were exposed to CS for 8 weeks using an exposure chamber system to mimic smoking conditions. Histomorphological analyses on muscle fibre type and cross‐sectional area were determined in soleus and extensor digitorum longus (EDL). Transcriptomic profiles were investigated for identifying differentially expressed genes **(**DEGs) and potential mechanistic pathways involved. Inflammatory responses in terms of the macrophage population and the level of inflammatory cytokines were measured. Markers for muscle‐specific proteolysis were also examined.

**Result:**

Soleus muscle, but not in EDL, exhibited a significant increase in Type IIa fibres (SA: 9.0 ± 3.3%; CS: 19.8 ± 2.4%, *p* = 0.002) and decrease in Type I fibres (SA: 90.1 ± 3.6%; CS: 77.9 ± 3.3%, *p* = 0.003) after CS exposure. RNA sequencing revealed 165 identified DEGs in soleus including upregulation of ‘Cd68’, ‘Ccl2’ and ‘Ucp2’ as well as downregulation of ‘Ucp3’, etc. Pathways enrichment analysis revealed that the upregulated pathways in soleus were related to immune system and cellular response, while the downregulated pathways were related to oxidative metabolism. Only 10 DEGs were identified in EDL with less enriched pathways. The soleus also showed elevated pro‐inflammatory cytokines, and the total macrophage marker CD68 was significantly higher in soleus of CS compared to the SA group (CD68^+^/no. of fibre: SA = 60.3 ± 39.3%; CS = 106.5 ± 27.2%, *p* = 0.0039), while the two groups in EDL muscle showed no significant difference. The expression of E3 ubiquitin ligase atrogin‐1 associated with muscle degradation pathways was 1.63‐fold higher in the soleus after CS, while no significant differences were observed in the EDL.

**Conclusion:**

The CS‐induced inflammatory responses on soleus muscle are likely mediated via targeting mitochondrial‐related signalling, resulting in mitochondrial dysfunction and impaired oxidative capacity. The presumably less active mitochondrial‐related signalling in EDL renders it less susceptible to changes towards CS, accounting for differential impacts between muscle types.

## 1 Introduction

Cigarette smoking is a well‐known hazard to health, increasing the incidence and mortality of chronic diseases. Over 7 million deaths result from tobacco smoking every year, and most are from cigarette [1]. Cigarettes contain several toxic chemicals, including nicotine and tar that are known to trigger an inflammatory response in the lungs [2]. Apart from being a major risk factor for various types of chronic diseases in the respiratory system, cigarette smoking is also associated with increased risks to other extrapulmonary systems, such as skeletal muscle [3].

Cigarette smoking is known as a risk factor contributing to decreases in exercise capacity and physical performance, which is believed to be due to cigarette smoke (CS)‐induced airway obstruction that limits ventilation and thus lowers exercise capacity [4, 5]; however, current studies have indicated that smokers can manifest exercise intolerance without overt pulmonary disorders. Some studies suggested that skeletal muscle weakness and reduced fatigue resistance were found in asymptomatic human smokers without known lung diseases [6, 7]. This suggests that exposure to smoking per se may contribute to musculoskeletal dysfunction prior to the occurrence of detectable pulmonary impairment. One plausible reason smoking reduces muscle fatigue resistance and tolerance may be due to a shift of fibre type from slow‐twitch fibres to fast‐twitch fibres in smokers [8]. Systematic reviews showed that slow‐twitch muscle exhibited significant oxidative‐to‐glycolytic fibre conversions in patients with COPD [9]. Similar findings in animal models were also summarised in a recently published review [10]. Yet, the extent of fibre type conversion could be dependent on multiple factors such as the species of animal used, duration and concentration of CS exposure and the types of muscle investigated. [10]. For instance, a significant fibre type conversion was observed in soleus but NOT in other fast‐twitch muscles such as extensor digitorum longus (EDL), plantaris and tibialis after CS exposure [10]. An animal study conducted by our group previously showed that after an acute period of CS exposure, a significant reduction in the proportion of slow‐twitch fibres, with a concomitant increase in the proportion of fast‐twitch fibres, was displayed in the soleus muscle in the absence of noticeable lung function impairment and that the changes were found to be dose‐dependent [11]. Consistently, the EDL muscle, a representative predominantly fast‐twitch‐fibre muscle, did not exhibit fibre type shifting after CS exposure.

Despite the well‐known discrepancy in fibre type and metabolic enzymes between the two muscle types, the mechanism underlying the susceptibility of slow muscles but not fast muscles after CS exposure is still unclear, thus hindering the development of therapeutical approach towards this extrapulmonary hazard.

The present study aimed to understand the discrepancies between fast‐twitch and slow‐twitch muscle fibres with regard to their sensitivity towards cigarette exposure. The soleus and EDL were chosen for this study owing to the predominantly slow‐twitch nature of the soleus and the fast‐twitch nature, with a diverse muscle fibre type composition, of the EDL. Data on gene expression differences between muscle types offer a deeper understanding of musculoskeletal impact induced by CS. RNA sequencing (RNA‐Seq) was used to assess the transcriptomic profiles and thus allowed for a quantitative comparison between the expression levels of genes in both slow‐twitch and fast‐twitch muscle in the presence or absence of CS. The result of these differential gene expression patterns at the transcriptome level could provide additional information that helps in understanding the mechanism causing fibre type shifting in muscle.

## 2 Methodology

### 2.1 Experimental Grouping and Animal Preparation

Twelve male Sprague–Dawley rats (6 weeks old) were randomly allocated into two groups: a CS group (CS: *n* = 6) and a sham air (SA) control group (SA: *n* = 6). The rats were obtained from the centralised animal facilities (CAFs) at the Hong Kong Polytechnic University and were housed in groups of two or three under a 12‐h light–dark cycle. They had free access to a standard laboratory rat diet and water. The temperature and relative humidity in the CAFs were controlled at 21°C ± 2°C and 60%, respectively. All experimental procedures were approved by the Animal Subjects Ethics Sub‐Committee of the Hong Kong Polytechnic University (ASESC No. 16‐17/26‐RS‐R‐GRF).

### 2.2 CS Exposure Model

The rats were exposed to either CS (i.e., the CS group) or SA group in an exposure chamber system [11] for 8 weeks to mimic a chronic‐CS‐exposure‐induced COPD‐like model [12]. Briefly, the exposure chamber apparatus consisted of two peristaltic pumps, 20L exposure chambers and a CEL‐712 Microdust Pro Real‐time Dust Monitor. Camel cigarettes (10 mg of tar, 0.8 mg of nicotine; R.J. Reynolds, Winston‐Salem, NC, USA) with their filters removed were used in our CS model. Fresh air (960 mL/min) and CS air (40 mL/min) were simultaneously pumped into the exposure chamber. A CEL‐712 Microdust Pro Real‐time Dust Monitor (Casella, UK) continually recorded the concentration of total particulate matter (TPM). The CS was kept at a concentration of TPM in 2.5 g/m^3^ [13]. In contrast, the SA group was exposed to fresh air only (1000 mL/min). The exposure time was 1 h per session, with two sessions per day for eight consecutive weeks.

### 2.3 Body Weight and Muscle Mass

The rats were weighed every week during CS. The hindlimb muscles, including the soleus and EDL, were isolated from rats under euthanisation via a sodium pentobarbitone overdose (i.p., 100 mg/kg). The muscle mass was measured via a four‐digit balance.

### 2.4 Muscle Fibre Size and Proportion of the Muscle Fibre Type

To evaluate the fibre type composition of the soleus and EDL after 8 weeks of CS exposure, identification of muscle fibre types (I, IIa and IIb) was conducted using multicolor immunofluorescence staining, which was shown to be a more precise and efficient method for characterizing both pure and hybrid fibres as compared to ATPase staining [14, 15]. The soleus and EDL muscle of the left legs were carefully isolated and embedded in an O.C.T.™ medium under liquid nitrogen. Cross‐sections that were 7 μm thick were cut from the muscle in the midbelly. The sections were air‐dried, fixed in ice‐cold acetone, blocked with 10% normal goat serum and incubated with specific primary and secondary antibodies (Table S1). The immunostained images were captured using SPOT Advanced software (Diagnostic Instruments Inc., USA). A total of eight frames of images covering both superficial and deep layers were randomly captured from muscle sections to minimise the selection bias. The total number of fibres from the captured images (approximately 125 fibres per frame) were counted for the fibre type distribution, and the data were presented as the percentage of type of fibre/total fibre counted. The cross‐sectional area (CSA) of the myofibers was determined. A total of 400 fibres per muscle (i.e, 8 frames × 50 fibres/frame) were analysed per sample [16].

### 2.5 Transcriptomic Analysis and Pathway Enrichment

Total RNA from the same muscles of the contralateral hindlimb (*n* = 3 per group) was isolated from the muscle tissue using an SV Total RNA isolation system extraction kit (Promega Corporation, USA), and purified RNA samples then underwent RNA sequencing. Raw sequencing data were subjected to quality control and the filtered clean reads were aligned to the reference sequence. The average alignment ratio of the sample comparison genome was 95.31%. After the QC and alignment, the quantification analysis was performed based on gene expression with the DNBSEQ platform. The RNA samples were subjected to differential expression gene (DEG) analysis using an online platform (https://biosys.bgi.com/). Gene functional enrichment analysis was also carried out via the use of the online BGI analysis platform. Gene Ontology (GO) and the Kyoto Encyclopedia of Genes and Genomes (KEGG) are genetic function databases that identify functional information (e.g. biological pathways) [17]. With these, significant DEGs were identified in the STRING database (http://string‐db.org) in order to evaluate the relationships of DEGs through protein–protein interaction (PPI) information. The information on protein interaction relationships was imported into Cytoscape software [18], which calculated the relationships between DEGs encoding proteins. Molecular complex detection (MCODE) was used to select PPI network modules in Cytoscape. Subsequently, gene set enrichment analysis (GSEA) was used to examine the significant regulatory changes in transcriptomic level between the SA and CS groups. The identified pathways of GSEA were presented with a normalised enrichment score (NES). A positive NES value indicated that the biological pathway was activated in the CS group when compared to the SA group, while a negative value indicated lower expression in the CS group [19].

### 2.6 RNA Reverse Transcription and qPCR (RT‐qPCR)

Following the same protocol described above, the total RNA was isolated from the rat muscle tissue (*n* = 6 per group) using SV Total RNA isolation system (Promega Corporation, US, Cat. Z3101). cDNA was synthesised from the extracted RNA using the GoScript™ Reverse Transcriptase Kit (Promega Corporation, USA, Cat. A5003). cDNA amplification was conducted using a CFX Connect Real‐Time PCR Detection System (Bio‐Rad Laboratories, USA) with specific probes and primers. The ΔΔCt method was employed for data analysis and normalisation of all gene expression data to the housekeeping gene (Hprt1, Ap3d1). Stable expression of the housekeeping gene was used as an internal control in each experiment to ensure consistent and reliable qPCR results. All primers used in this study were validated by examining their melt curves and peaks, confirming target specificity for each PCR reaction. The primer sequences are listed in Table S2.

### 2.7 Inflammatory Response Marker

Sections of the soleus and EDL were immunostained with antibodies against the macrophage‐specific marker CD68 (Table S1) with same procedure as for MHC staining. The number of individual muscle fibre types and positive CD68 cells were counted using ImageJ software. The protein levels of pro‐inflammatory cytokine TNF‐**α** and IL‐1β were evaluated using enzyme‐linked immunosorbent assay (ELISA) (Invitrogen Thermo Fisher Scientific, Monza, Italy, Cat. #ERA57RB and #BMS630).

### 2.8 Western Blotting of Muscle Degradation‐Related Protein

The protein samples from muscle tissue samples were extracted with an ice‐cold radioimmunoprecipitation assay (RIPA) lysis buffer. The sodium dodedyl‐sulfate‐polyacrylamide gel for total protein normalisation was prepared using TGX Stain‐Free FastCast Acrylamide Kit (BioRad Laboratories, USA). The separated β‐mercaptoethanol‐denatured protein samples were transferred to PVDF membrane using Western blotting. The total protein loaded on the membrane was scanned and captured by a ChemiDoc Imaging System (Bio‐Rad Laboratories, USA) for subsequent normalisation. Afterwards, the membrane was skim milk‐blocked before incubating with appropriate primary and secondary antibodies (Table S1). Subsequently, a chemiluminescence reaction was developed using Clarity and Clarity Max ECL Western Blotting Substrates kits (Bio‐Rad Laboratories, USA), and the images were captured. Image Lab software (Bio‐Rad Laboratories, USA) was used to quantify the level of signal intensities.

### 2.9 Statistical Analyses

Data are presented as the mean ± standard error of the mean (SEM) or percentage with interquartile range. Data were analysed via IBM® SPSS® statistics (Version 23.0, Chicago, IL, USA). An independent *t*‐test was used to detect the between‐group differences in the changes in the muscle fibres between the SA and CS groups. The level of significance was set at 0.05. For the transcriptomic data, significant DEGs were identified by < 0.05 of adjusted *p*‐values for multiple testing, known as the (*Q*‐value). For the functional enrichment analysis and GSEA analysis, an enrichment cutoff was applied at the NES (NES > 1) and false discovery rate (FDR < 0.05).

## 3 Results

### 3.1 Body Weight and Muscle Mass

After 8 weeks of CS exposure, the CS group demonstrated a significant reduction in body weight (*p* = 0.011) and muscle mass in both the soleus (*p* < 0.001) and EDL (*p* < 0.001) relative to the SA group (Table 1). When normalised by body weight, a significantly lower normalised muscle mass in the CS group versus the SA group was demonstrated in the soleus muscle (*p* = 0.041) but not the EDL.

**TABLE 1:** The relative change of the body weight and muscle mass before and after the 56‐day cigarette smoke (CS) exposure.

		SA	CS
BW (g)	Pre	267 ± 5	264 ± 5
	Post	453 ± 12	408 ± 8*
BW changes (%)		69 ± 2	55 ± 4*
MM (mg)	Soleus	226 ± 6	179 ± 5*
	EDL	201 ± 5	165 ± 3*
MM/BW (mg/g)	Soleus	0.5 ± 0.02	0.44 ± 0.01*
	EDL	0.45 ± 0.02	0.41 ± 0.01

*Note:* Data are presented as means ± SEM.

Abbreviations: BW, body weight; CS, cigarette smoke; MM, muscle mass; SA, sham air.

* *p* < 0.05, and

** *p* < 0.01.

### 3.2 Proportion of Muscle Fibre Type Distribution

**FIGURE 1:**
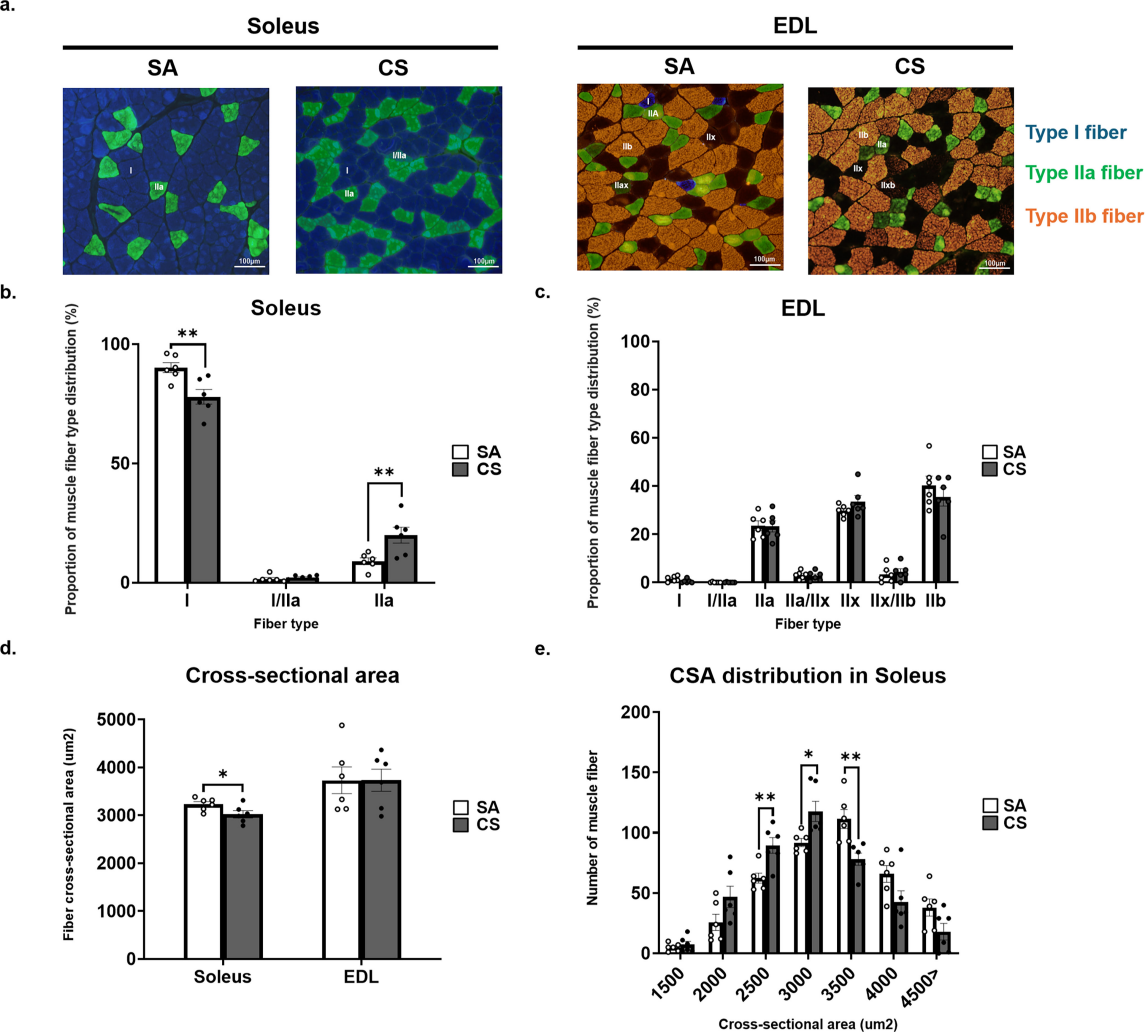
Effect of cigarette smoke (CS) exposure on the fibre types and cross‐sectional area (CSA) of the soleus and extensor digitorum longus (EDL) muscles. (a) Immunofluorescent staining of myosin heavy chain: Type I (blue); Type IIa (green); Type: IIb (brown); and Type IIx (colourless). Scale bars: 100 μm. (b) Number and proportion of fibre types in soleus. (c) Number and proportion of fibre types in EDL. (d) CSA of total muscle fibre in soleus and EDL. (e) CSA distribution in soleus. Images are representative of *n* = 6 rats per group. Data are expressed as mean ± standard error of mean (SEM). Statistical significance between Sham Air (SA) and CS groups was determined by independent *t*‐test. * indicates *p* < 0.05, ** indicates *p* < 0.01 between SA and CS groups.

In Figure 1b, the proportion of type 1 muscle fibres in the SA group and CS group was 90.1 ± 3.6% and 77.9 ± 3.3%, respectively, with a significant between‐group difference detected (mean difference = 12.2%, *p* = 0.003). For Type IIa fibres, the CS group (19.8 ± 2.4%) also showed a significantly higher proportion than the SA group (9.0 ± 3.3%), by 10.8% (*p* = 0.002) (Figure 1b). In contrast, no between‐group difference was detected in Type I/IIa hybrids (Figure 1b). As for the EDL muscle, the majority of the muscle fibres were predominantly fast‐twitch muscle fibres; however, there was no statistical difference in any of the fibre types shown in the EDL in the CS group as compared to those of the SA group (IIa: 23.3 ± 2.3% in SA vs. 23.6 ± 2.0 in CS, *p* = 0.932; IIx: 33.6 ± 2.5% in SA vs. 29.8 ± 0.9% in CS, *p* = 0.192; and IIb: 35.5 ± 3.8% in SA vs. 40.2 ± 3.9% in CS, *p* = 0.401; Figure 1c).

### 3.3 CSA of Muscle Fibre

After 8 weeks of CS exposure, the CSA of myofiber was significantly lower than that in the SA group in soleus (*p* = 0.045), while no between‐group difference was detected in EDL (*p* = 0.998). The CSA distribution of the soleus is shown in Figure 1e. There was a shift to the left in the fibre size distribution curve in the soleus muscle of the CS group when compared with the SA group (Figure 1e). Specifically, smaller fibres (≤ 3500 μm^2^) were more abundant in the CS group, while larger fibres (≥ 3500 μm^2^) were more abundant in the SA group (Figure 1e).

### 3.4 Differentially Expressed Gene (DEG) Analysis

**FIGURE 2:**
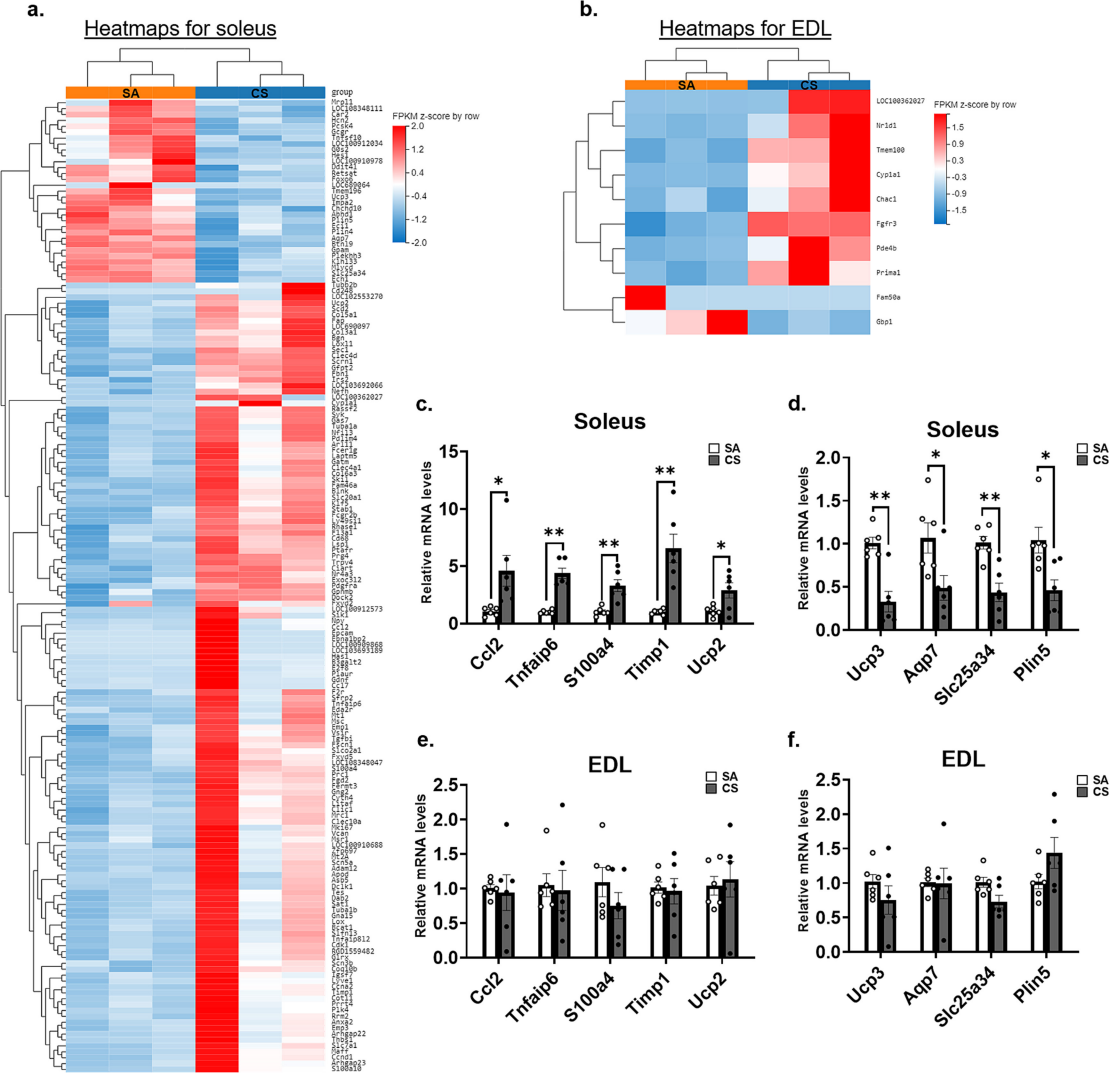
Differentially expressed genes (DEGs) between sham Air (SA) and cigarette smoke (CS) groups (*n* = 3). (a) Heatmaps presented differentially expressed genes between SA and CS groups in soleus. (b) Heatmaps presented differentially expressed genes between SA and CS groups in extensor digitorum longus (EDL). RT‐qPCR validation of selected DEGs from soleus (c‐d) (*n* = 6) with (c) indicated upregulated genes and (d) indicated downregulated genes and same selected DEGs examined from EDL (e, f) (*n* = 6) with (e) indicated upregulated genes and (f) indicated downregulated genes. Data are expressed as mean ± standard error of mean (SEM). Statistical significance between SA and CS groups was determined by independent *t*‐test. * indicates *p* < 0.05, ** indicates *p* < 0.01 between SA and CS groups.

We compared gene expression levels between the CS and SA groups to identify the upregulated/downregulated DEGs. In the soleus, we identified 17 503 genes. Of these, 165 DEGs (upregulated: 134 DEGs; downregulated: 27 genes) were found to be significantly different between the CS and SA groups (Figure 2a and S1). Upregulated DEGs, such as ‘Cd68’, ‘Ccl2’, ‘F2r’ and ‘Timp1’, were associated with the regulation of inflammation and oxidative stress response in muscle tissue. There were DEGs associated with cellular processes, including the downregulation of ‘G0s2’ and ‘FoxO6’, as well as the upregulation of ‘Gas7’, ‘Ccnd1’ and ‘Irs2’. Some DEGs were also related to metabolic regulation, e.g., reduced levels of ‘Ucp3’, ‘Plin5’ and ‘Eci1’. As for the EDL, only 10 out of 16 613 genes demonstrated significant between‐group differences (Figure 2b). Table S3 shows the expression levels of the DEGs. To confirm the results of the RNA sequencing data, RT‐qPCR analysis was performed. The results confirmed that inflammatory‐related transcripts, including Timp1, Ccl2, Tnfaip6 and S100a4, were significantly upregulated in the CS soleus muscle (*p* < 0.05) (Figure 2c). Conversely, mitochondrial‐related DEGs, such as Ucp3, Plin5 and Slc25a34, were significantly downregulated in the CS soleus muscle (Figure 2d) but not in the EDL muscle (Figure 2e–f).

### 3.5 Identification of Hub Genes and Modular Screening

The STRING online database was used to identify the PPI network of DEGs. In the soleus, a total of 159 nodes and 234 edges were obtained with a scoring value > 0.4 (Figure S2). Cytoscape software was then used to identify hub genes in the PPI network of the soleus. The top 10 hub genes in the soleus are listed in Table S4. Furthermore, an analysis of the EDL muscle was conducted to explore the PPI network approach; however, no edges or significant interactions were found in the EDL muscle.

### 3.6 GO Term Enrichment Analyses and KEGG Pathway Analyses

**FIGURE 3:**
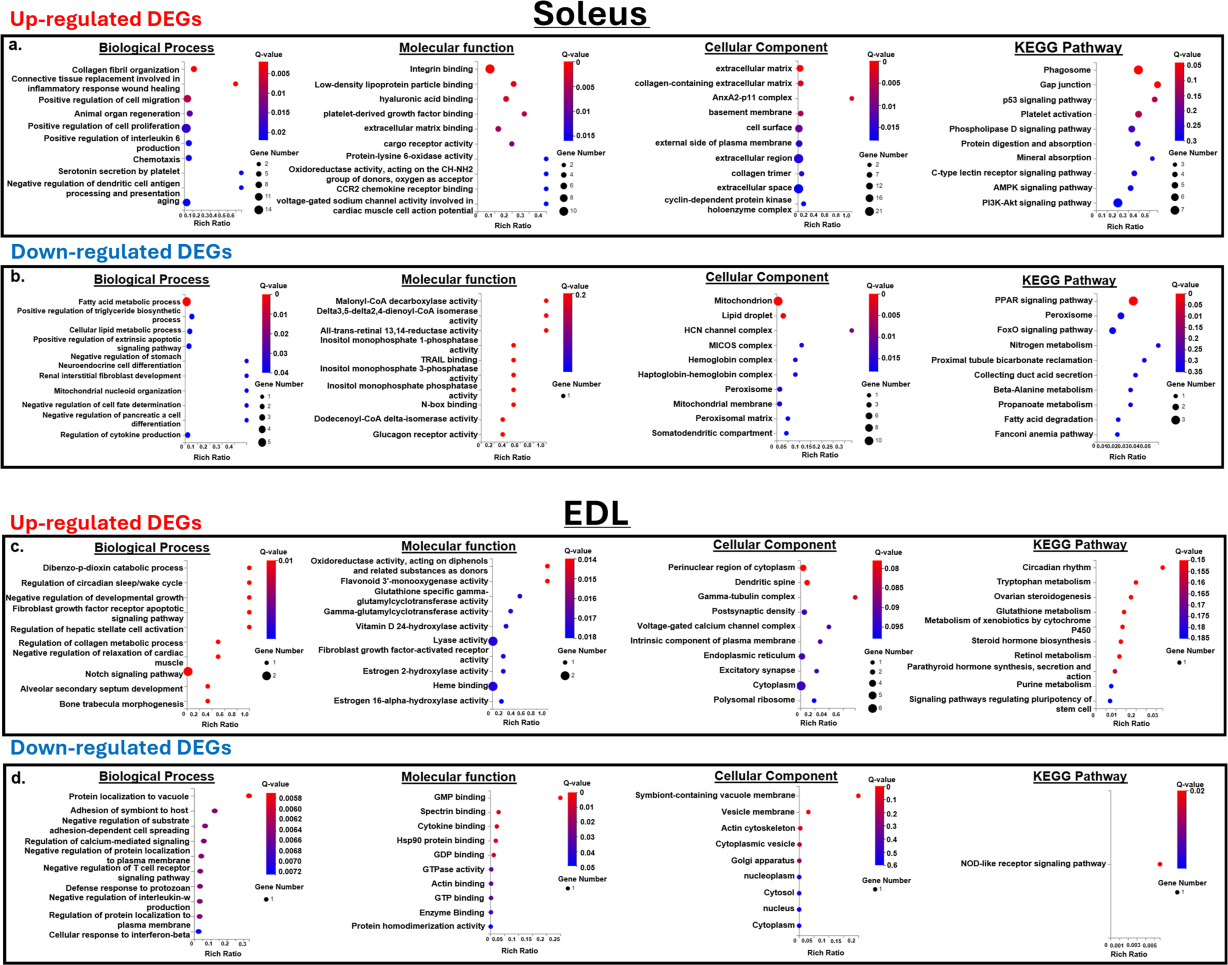
GO and KEGG pathway enrichment analysis of differentially expressed genes (DEGs) in the soleus and EDL of cigarette smoke (CS) rats. The enrichment analyses included the GO biological process, molecular function, cellular components and KEGG pathway. The top 10 pathways of the (a) upregulated DEGs and (b) the downregulated DEGs in the soleus between sham air (SA) and CS rats; the top 10 pathways of the (c) upregulated DEGs and (d) the downregulated DEGs in the EDL between SA and CS rats.

The top 10 GO terms identified by GO enrichment analysis of the DEGs are presented in Figure 3. In the soleus, the upregulated genes identified were related to biological processes like collagen fibril organisation, connective tissue replacement in the inflammatory response and wound healing and interleukin 6 production. Other upregulated genes were enriched in integrin binding, hyaluronic acid binding and cellular components such as the extracellular matrix, cell surface and extracellular region. On the other hand, the downregulated genes were associated with fatty acid or lipid metabolic processes and the cellular component terms mitochondrion and HCN channel complex. KEGG pathway enrichment analysis further revealed that the downregulated genes were involved in the PPAR signalling pathway. For the EDL, the upregulated DEGs were involved in dibenzo‐p‐dioxin catabolic processes and the negative regulation of developmental growth, increased oxidoreductase activity and gamma‐glutamylcyclotransferase activity, and were enriched in the perinuclear region of the cytoplasm. Whereas the downregulated genes were involved in the biological process of protein localization to vacuoles and the adhesion of symbionts to the host. Additionally, these downregulated genes were also associated with decreased GMP binding. Notably, in the KEGG pathway analysis, the downregulated genes were associated with the NOD‐like receptor signalling pathway.

### 3.7 Function and Enrichment Analysis of GSEA

**FIGURE 4:**
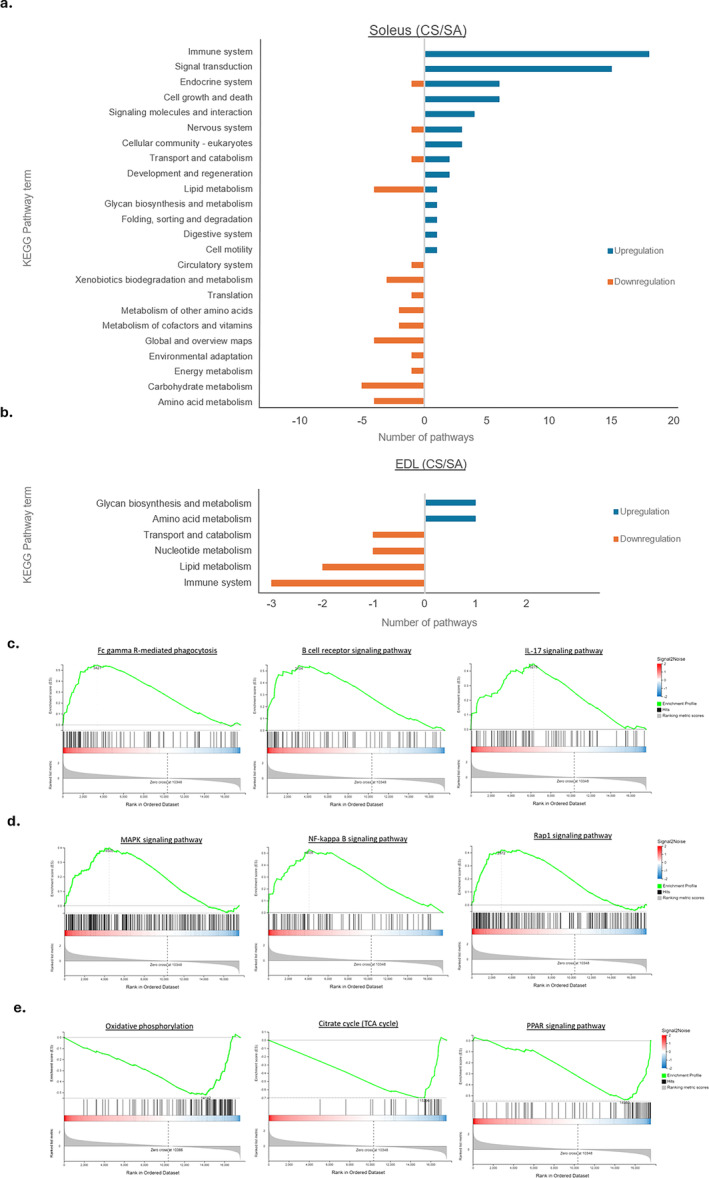
The results of gene set enrichment analysis (GSEA). (a) Results of GSEA in soleus and (b) EDL when compared between sham air (SA) and cigarette smoke (CS). Panels (c–e) indicate an example of an enrichment plot of GSEA results from the KEGG database in the soleus. The green line in the GSEA graph represents the enrichment score (ES) across the ranked list of genes. The ES indicates the degree to which a gene set is overrepresented at the top or bottom of the ranked list of genes. A positive ES indicates upregulated pathways, while a negative ES indicates downregulated pathways. (c) Upregulation of the immune system‐related pathways. (d) Upregulation of signal transduction‐related pathways. (e) Downregulation of metabolic‐related pathways. Left side of the enrichment plot (red colour side) indicates CS group, while right side of the enrichment plot (blue colour side) indicates SA group.

GSEA was used to analyse the potential biological regulation of functions and signalling pathways from the total defined set of expression gene results in the soleus and EDL after CS exposure. Based on the KEGG database, it was found that the soleus and EDL were enriched with several critically regulated pathways after CS exposure, using an enrichment cutoff (NES > 1 and FDR < 0.05) (Tables 2 and S5). Specifically, the soleus was identified as having 95 pathways within the enrichment cuffoff (Figure 4a), while the EDL showed only nine pathways (Figure 4b). Moreover, 64 out of the 95 identified pathways were shown to be upregulated in the soleus subjected to CS treatment when compared to SA. The top enriched KEGG term was related to the immune system, which had 18 pathways, among which the key pathways included ‘Fc gamma R‐mediated phagocytosis’, the ‘B cell receptor signaling pathway’, the ‘IL‐17 signaling pathway’ and so on (Figure 4c). The next KEGG category term was associated with signal transduction, involving the ‘NF‐kappa B signaling pathway’, the ‘MAPK signaling pathway’ and the ‘FoxO signaling pathway’, among others (Figure 4d). On the other hand, 31 identified pathways were suppressed in the soleus upon CS treatment when compared to SA, and most were related to metabolism, such as ‘oxidative phosphorylation’ and the ‘citrate cycle’, and the ‘PPAR signaling pathway’ was also inactivated (Figure 4e). In the EDL muscle, the results suggested a smaller number of enriched pathways under CS when compared to the soleus. The signalling pathways that were significantly activated in the EDL upon CS exposure were ‘Glycosylphosphatidylinositol (GPI)‐anchor biosynthesis’ and ‘Glycine, serine and threonine metabolism’, while a total of six pathways were significantly suppressed after CS treatment, including ‘Antigen processing and presentation’, ‘Pyrimidine metabolism’ and ‘Intestinal immune network for IgA production’. Interestingly, the two pathways ‘Antigen processing and presentation’ and ‘Intestinal immune network for IgA production’ were upregulated in the soleus but downregulated in the EDL muscle. According to the GO database of biological function, there were 154 upregulated and 31 downregulated pathways in the soleus in CS when compared to the SA group. Some of the upregulated pathways were categorised into ‘immune system’ and the ‘regulation of biological process’, while 21 GO biological process pathways were downregulated and they were associated with metabolism processes. These results were consistent with the findings from the KEGG database. Only seven pathways in the EDL muscle were dynamically altered after CS exposure, with six of them upregulated and one downregulated. Among the pathways that were altered by CS, the ‘Antigen processing and presentation of exogenous peptide antigen via MHC class II’ was suppressed in the EDL muscle but activated in the soleus.

**TABLE 2:** The top 10 significantly enriched GGSEA results of the soleus and EDL muscle.

ID	Description	setSize	NES	FDR
Pathway—Soleus (upregulated)				
04520	Adherens junction	70	2.091	< 0.001
04380	Osteoclast differentiation	117	2.006	< 0.001
04666	Fc gamma R‐mediated phagocytosis	94	1.966	0.001
04650	Natural killer cell mediated cytotoxicity	85	1.949	0.001
04662	B cell receptor signalling pathway	75	1.906	0.001
04115	p53 signalling pathway	72	1.909	0.001
04064	NF‐kappa B signalling pathway	93	1.898	0.002
04625	C‐type lectin receptor signalling pathway	100	1.871	0.002
04611	Platelet activation	122	1.863	0.002
04062	Chemokine signalling pathway	164	1.841	0.002
Pathway—Soleus (downregulated)				
190	Oxidative phosphorylation	120	−2.940	< 0.001
4146	Peroxisome	80	−2.798	< 0.001
20	Citrate cycle (TCA cycle)	29	−2.728	< 0.001
280	Valine, leucine and isoleucine degradation	52	−2.657	< 0.001
640	Propanoate metabolism	30	−2.546	< 0.001
3320	PPAR signalling pathway	72	−2.478	< 0.001
71	Fatty acid degradation	44	−2.424	< 0.001
630	Glyoxylate and dicarboxylate metabolism	28	−2.362	< 0.001
650	Butanoate metabolism	25	−2.231	< 0.001
1200	Carbon metabolism	107	−2.230	< 0.001
Pathway—Extensor digitorum longus (upregulated)				
563	Glycosylphosphatidylinositol (GPI)‐anchor biosynthesis	26	2.025	0.008
260	Glycine, serine and threonine metabolism	34	1.828	0.041
Pathway—Extensor digitorum longus (downregulated)				
4612	Antigen processing and presentation	65	−1.905	0.005
4672	Intestinal immune network for IgA production	31	−1.862	0.008
240	Pyrimidine metabolism	49	−1.819	0.010
120	Primary bile acid biosynthesis	15	−1.829	0.010
4146	Peroxisome	77	−1.776	0.016
71	Fatty acid degradation	41	−1.723	0.026
4640	Haematopoietic cell lineage	74	−1.665	0.037

*Note:* NES with positive value: upregulated in the treatment group (CS group); NES with negative value: downregulated in the treatment group.

Abbreviations: FDR, false discovery rate; NES, normalised enrichment score.

### 3.8 Inflammatory Response in the Soleus and EDL

**FIGURE 5:**
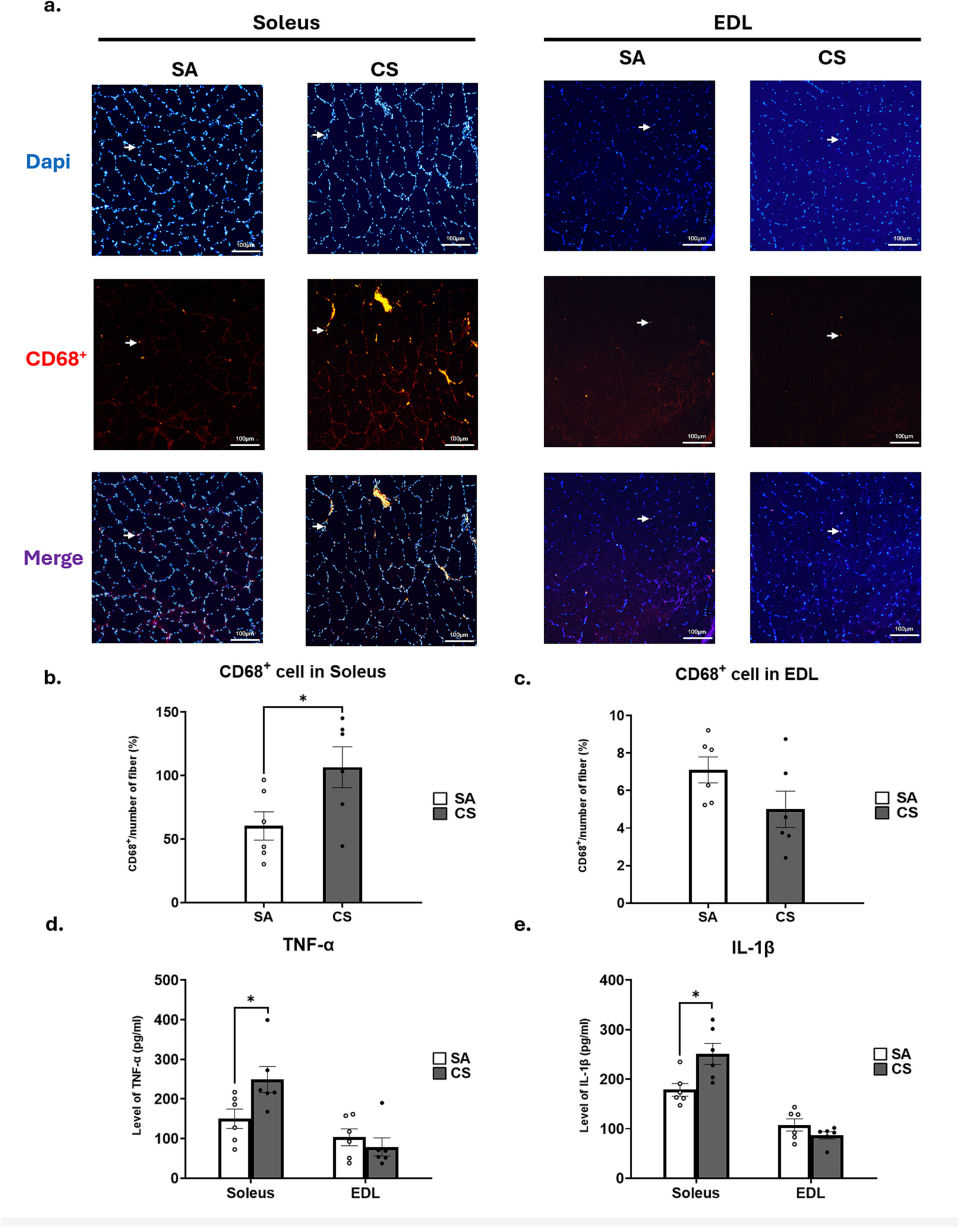
The inflammatory response of the soleus and EDL muscles after cigarette smoke (CS) exposure. (a) Immunofluorescent staining of CD68^+^ cell and total nucleus (DAPI) on soleus and extensor digitorum longus (EDL). The blue colour indicates DAPI; the red colour indicates CD68^+^; the white arrow pinpoints the CD68^+^ cells. Scale bars: 100 μm. (b) Relative cell count of CD68^+^ cell/number of fibres in soleus. (c) Relative cell count of CD68^+^ cell/number of fibres in EDL. (d) Quantitation of level of TNF‐α in soleus. (e) Quantitation of level of TNF‐α in EDL. Graphs are representative of *n* = 6 rats per group. Data are expressed as mean ± standard error of mean (SEM). Statistical significance between sham air (SA) and cigarette smoke (CS) groups was determined by independent *t*‐test. * indicates *p* < 0.05 between SA and CS groups.

In the soleus, the expression of CD68‐positive cells was about twofold higher in the CS group than that in the SA group (Figure 5b), while no between‐group differences were detected in the EDL (Figure 5c). Similarly, the CS group evoked an inflammatory response, as reflected by a significant increase in tumour necrosis factor‐alpha (TNF‐α) and interleukin l‐beta (IL‐1β) in the soleus (TNF‐α, *p* = 0.036; IL1 β, *p* = 0.044), but no detectable changes were observed in the EDL (Figure 5d,e).

### 3.9 Muscle Degradation‐Related Protein

**FIGURE 6:**
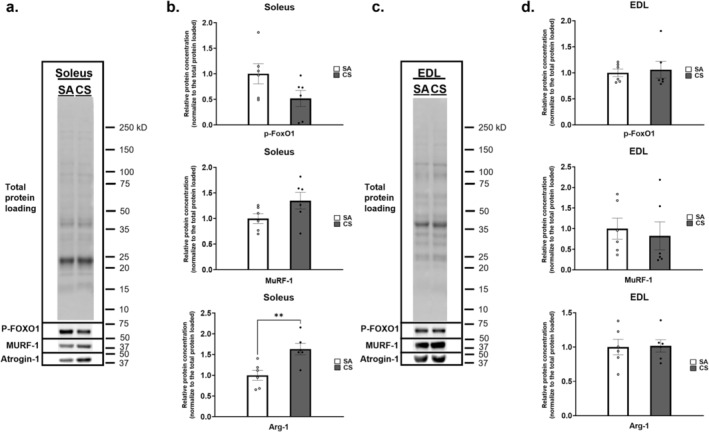
The protein levels of Atrogin‐1, MuRF‐1 and phosphorylation FoxO1 (p‐FoxO1). (a) Immunoblot analysis of the protein levels of Atrogin‐1, MuRF‐1 and p‐FoxO1 in soleus between the sham air (SA) and cigarette smoke (CS) groups; representative immunoblots are shown. (b) Quantities of the fold change in the expression level of p‐FoxO1, MuRF‐1 and Atrogin‐1 in soleus. (c) Immunoblot analysis of the protein levels of Atrogin‐1, MuRF‐1 and p‐FoxO1 in extensor digitorum longus (EDL) between the SA and CS groups. (d) Quantities of the fold change in the expression level of p‐FoxO1, MuRF‐1 and Atrogin‐1 in EDL. The protein intensities were normalised to the total protein loaded. Graphs are representative of *n* = 6 rats per group. Data are expressed as the mean ± standard error of mean (SEM). Statistical significance between SA and CS groups was determined by independent *t*‐test. ** indicates *p* < 0.01 between the SA and CS groups.

In accordance with the identification of activation of the ‘NF‐kappa B signaling pathway’ and the ‘FoxO signaling pathway’, we examined the expression level of two skeletal muscle‐specific proteolytic markers, E3 ubiquitin ligases atrogin‐1 and MuRF‐1, to determine if FoxO1 phosphorylation‐dependent protein degradation was active in the soleus and EDL during CS exposure. The relative protein concentrations were normalised to the total protein loaded. Though insignificant, the protein levels of phospho‐FoxO1 in soleus showed a decreasing trend by twofold, while MuRF‐1 in the soleus showed an increasing trend of 1.55‐fold, after CS exposure (Figure 6a,b). Nonetheless, the level of atrogin‐1 in soleus was significantly increased by 1.63‐fold in CS group (Figure 6d). Yet, no significant between‐group differences were detected in the expression levels of phospho‐FoxO1, atrogin‐1 and MuRF‐1 in the EDL (Figure 6c,d).

## 4 Discussion

We have previously demonstrated oxidative‐to‐glycolytic fibre type shifting in the soleus muscle after acute CS exposure [11], while the fast‐twitch EDL muscle was resistant to CS exposure. In our present chronic 8‐week CS exposure model, the soleus muscle exhibited changes in fibre type proportions which may indicate muscle metabolic impairment, whereas the reduction in muscle CSA may be associated with muscle atrophy. These changes suggested muscle impairment in the soleus muscle after CS [20, 21]. Despite prolonged exposure, the EDL muscle was still insensitive towards CS, which has also been reported in other studies [10]. Although numerous studies have already reported that slow‐ and fast‐twitch muscles differ in many aspects [22, 23], the differential responses between the two types of muscles to CS that have been reported are only confined to muscle mass, CSA and fibre type composition, whereas the molecular changes and the underlying mechanisms are largely unclear.

Based on the results of our transcriptomic analysis, the differential gene expression patterns between the soleus and EDL muscles have diverse regulatory effects on biological processes and signalling pathways under smoking conditions. The soleus exhibited a more than 10‐fold higher number of enriched pathways and DEGs than the EDL after CS exposure, indicating a very different response between the two muscle types towards CS. We identified the top 10 hub genes in the soleus, all of which involve major responses in the tissue inflammation process or tissue remodelling. From the result of GSEA under the KEGG database, several immune‐related pathways were activated in the soleus after CS. For example, the ‘IL‐17 signaling pathway’, ‘Chemokine signaling pathway’ and ‘B cell receptor signaling pathway’ were indicative of the early phase of the inflammatory response in the soleus after smoking. From the DEG analysis, an increase in the expression of ‘Fcgr2b’, ‘Ccr7’ and ‘Cd68’ in the soleus after CS has also been revealed. Fcgr2b encodes for Fc gamma receptor IIB, which is the only inhibitory Fc receptor controlling various inflammatory responses [24]. Our GSEA result also highlighted ‘Fc gamma R‐mediated phagocytosis’, ‘Lysosome’ and ‘phagosome’, which suggested the involvement of macrophages in the soleus upon CS exposure. Ccr7 encodes for a membrane receptor that is exclusively expressed on the cell surface of M1 macrophages [25]. Meanwhile, the top 10 hub genes analysis found that ‘Ccl2’ and ‘Cd68’ play a critical role as connectors within the biological network. Ccl2 acts as a chemoattractant in the recruitment and activation of monocytes as well as macrophages to sites of inflammation [26], while the Cd68 gene encodes the macrophage‐specific marker CD68 which is highly expressed in M1 macrophages [27]. An immunohistochemical analysis, indicating a significantly higher number of CD68‐positive cell in soleus, supported the notion of monocyte/macrophage activation after CS [28]. The macrophage activation is likely mediated by the elevated release of TNF‐α and IL‐1β, as detected via ELISA. Subsequently, lysosomal proteases and the release of reactive oxygen species (ROS) play a crucial role in phagosome digestion [29].

The enrichment analysis in the GO database yielded similar results, indicating the activation of immune‐related biological processes such as ‘phagocytosis’, ‘B cell receptor signaling pathway’ and ‘T cell activation’, as well as the production of IL‐6 and IL‐12. These processes suggest that CS amplifies a localised inflammatory response in the soleus muscle that leads to tissue damage. The upregulation of the signalling pathways such as PI3K‐Akt, Rap1 and p53 signalling all involved in cell survival and regeneration and may reflect the soleus muscle's attempt to adapt to the stress induced by smoking.

Prolonged inflammation in the soleus disrupts the balance between cell survival and damage [30], which leads to mitochondrial damage as well as dysfunction and eventually altered oxidative metabolism, as reflected by the downregulation of oxidative metabolism pathways such as ‘Citrate cycle (TCA cycle)’ and ‘Oxidative phosphorylation’, shown in our enrichment analysis. These pathways are vital for energy generation in the muscle [31]. Their downregulation suggests impaired ATP and NADH production as well as utilisation [32], potentially affecting muscle contractions and exercise performance [9]. Unlike human quadriceps, in which Type I fibres are more oxidative than Type IIa fibres, the Type IIa fibres in rat soleus expressed a comparatively higher content of succinate dehydrogenase than its Type I counterpart [33]. It is believed that the Type I‐to‐IIa conversion observed in the CS model is a compensatory mechanism in response to CS‐induced hypoxia. However, our transcriptomic data indicated that the oxidative pathways in CS were compromised, suggesting that the mechanism failed to compensate. Similar results have been reported by Decker and his colleagues, suggesting that CS directly impairs pyruvate‐stimulated mitochondrial respiration, which is the primary mechanism underlying CS‐induced mitochondrial dysfunction [34].

Furthermore, our DEG data in the CS‐exposed soleus revealed the dynamic expression of two members of the mitochondrial uncoupling protein (UCP): a 3.0‐fold downregulation of ‘Ucp3’ accompanied by a 2.9‐fold upregulation of ‘Ucp2’. UCP3 knockout mice exhibited an increase in ROS production in the skeletal muscle mitochondria [35], while an increase in ‘Ucp2’ expression suggests that the mitochondria within the muscle limit ATP production and alter energy production in the soleus [36]; therefore, the mitochondrial metabolic changes are possibly a response to the suppressed oxidative metabolism of energy generation in the soleus. Additionally, mitochondrial dysfunction may contribute to muscle cell apoptosis or degradation. A higher expression of the muscle‐specific E3 ubiquitin ligases atrogin‐1 and MuRF1 was detected in the soleus following CS, suggesting that smoke exposure may stimulate the ubiquitin proteasome system and be involved in the muscle degradation process [37].

The results of this study support the notion of an altered muscle fibre type and metabolic phenotype in the soleus muscle after smoking. These results reflect the soleus muscle's attempt to adapt to smoking‐induced impact. The observed upregulation of pathways associated with cell and inflammation responses suggests the activation of adaptive mechanisms and potential tissue damage in the soleus muscle. It is noteworthy that the effects of CS on oxidative fibres are significant, as these fibres possess a high mitochondrial content. The downregulation of pathways associated with oxidative metabolism supports the potential impairment on mitochondrial function and decreases oxidative capacity in soleus. This potentially supports the mechanism contributing to increased muscle fatigue in smokers.

Meanwhile, the EDL appears to exhibit a completely different story. The DEGs in the EDL muscle showed much fewer inflammatory‐related responses compared to the soleus muscle. GSEA also revealed the downregulation of immune response pathways, specifically ‘Antigen processing and presentation’. These changes suggest that the EDL muscle may have a potential immune suppressive effect, or, in other words, a high threshold of the inflammatory response under CS conditions. Consequently, either such defaulted, inactive immune responses were unable to trigger inflammatory reactions or the ubiquitin proteosome appears to protect the muscles from CS‐induced damage.

The EDL muscle has a different metabolic profile, relying more on glycolytic metabolism and having a lower oxidative capacity. As a result, the impact of CS on mitochondrial function and oxidative capacity is less pronounced in the EDL. Interestingly, one study found that blood flow was in the order of fast‐twitch red (Type IIa) > slow‐twitch (Type I) > fast‐twitch white (Type IIb) [38]. This suggests that the relationship between fibre type and blood flow may differ between the EDL and soleus muscles [38, 39]. In our study, EDL appeared to have a greater proportion of Type IIb and IIx fibres, whereas soleus was primarily composed of Type I and Type IIa fibres suggesting the regional differences in blood supply. These fibres heavily rely on a robust blood supply to meet their high metabolic demands, making them vulnerable to systemic inflammatory responses. On the other hand, previous studies have also demonstrated that slow‐twitch muscle with a much higher mitochondrial content compared to fast‐twitch muscle is associated with higher ROS production and greater oxidative damage [40]. This may help explain why the glycolytic EDL muscle is less prone to oxidative damage.

## 5 Conclusion

Our findings elucidate an interesting phenomenon, in which slow‐ and fast‐twitch muscle displays a very different response towards CS in terms of histological, biochemical and transcriptomic profiles. To our understanding, the present study is the first of its kind to comprehensively compare the response of different muscle types to CS exposure. CS is likely to exert damaging effects on slow‐twitch muscle via various mitochondrial‐related pathways, which are, however, inactive in fast‐twitch muscle. These findings help explain the differential responses of slow‐twitch versus fast‐twitch muscles to CS in different studies that have been reported previously. Future studies are suggested to identify the specific genes and the pathways involved, which are responsible for the adverse effects of CS on muscle dysfunction.

## Conflicts of Interest

The authors declare no conflicts of interest.

## Supporting information


**Table S1** List of major antibodies and cocktail for Immunostaining and Western Blot Analysis.
Table S2. List of TaqMan™ Assays used in the present study. Manufacturer assay ID can be referred to as Thermo Fisher Scientific, USA.
Table S3. The list of the DEGs of the soleus and EDL muscle.
Table S4. Top10 hub genes identified in DEGs of soleus.
Table S5. List of the significantly enriched GSEA results of the soleus and EDL muscle.
Figure. S1 Volcano plots and Venn diagram of significantly expressed genes in the soleus and Extensor Digitorum Longus (EDL). (a) Volcano plots significantly expressed genes in the soleus (b) Venn diagram of gene intersection between the soleus and EDL (c) Volcano plots significantly expressed genes in the EDL. The colour of the dots in volcano plots indicates a gene with no significant difference (grey), that has been upregulated (red) and that has been downregulated (green).
Figure. S2 PPI networks of DEGs in the soleus. (a) A total of 165 DEGs were imported into the STRING online database and the PPI network was obtained with 234 edges.
