# Supplementary material for: Transcriptomic Profiling Reveals Differences in Slow‐Twitch and Fast‐Twitch Muscles of a Cigarette Smoke‐Exposed Rat Model
Source: J Cachexia Sarcopenia Muscle. 2024 Nov 29;16(1):e13633. doi: 10.1002/jcsm.13633 (PMC11670162; doi:10.1002/jcsm.13633)
Supplement: Supplementary file 1 — Table S1 List of major antibodies and cocktail for Immunostaining and Western Blot Analysis. Table S2. List of TaqMan™ Assays used in the present study. Manufacturer assay ID can be referred to as Thermo Fisher Scientific, USA. Table S3. The list of the DEGs of the soleus and EDL muscle. Table S4. Top10 hub genes identified in DEGs of soleus. Table S5. List of the significantly enriched GSEA results of the soleus and EDL muscle. Figure. S1 Volcano plots and Venn diagram of significantly expressed genes in the soleus and Extensor Digitorum Longus (EDL). (a) Volcano plots significantly expressed genes in the soleus (b) Venn diagram of gene intersection between the soleus and EDL (c) Volcano plots significantly expressed genes in the EDL. The colour of the dots in volcano plots indicates a gene with no significant difference (grey), that has been upregulated (red) and that has been downregulated (green). Figure. S2 PPI networks of DEGs in the soleus. (a) A total of 165 DEGs were imported into the STRING online database and the PPI network was obtained with 234 edges. [file JCSM-16-e13633-s001.docx]

Table S1. List of major antibodies and cocktail for Immunostaining and Western Blot Analysis

| Protein Target | Applications | Primary Antibodies Cocktails and Concentrations | Secondary Antibodies Cocktails and Concentrations |
| --- | --- | --- | --- |
| Myosin heavy chain I (MHC-I) | IF | BA-F8 (1:50) | Alexa Fluor 350 anti-mouse IgG2b (1:500) |
| Myosin heavy chain IIa (MHC-IIa) | IF | SC-71 (1:600) | Alexa Fluor 488 anti-mouse IgG1 (1:500) |
| Myosin heavy chain IIb (MHC-IIb) | IF | BF-F3 (1:100) | Alexa Fluor 555 anti-mouse IgM (1:500) |
| CD68 | IF | #97778 (1:200) | Alexa Fluor 488 anti-mouse IgG (1:500) |
| Muscle RING-finger protein-1 (MuRF1) | WB | #4305 (1:1000) | Anti-rabbit IgG-HRP SC-2357 (1:2500) |
| Atrogin-1 | WB | #30919 (1:1000) | Anti-rabbit IgG-HRP SC-2357 (1:2500) |
| Phospho-FoxO1 | WB | #84192S (1:1000) | Anti-rabbit IgG-HRP SC-2357 (1:2500) |

*Note:* IF= Immunofluorescence; WB = Western blot

Table S2. List of TaqMan™ Assays used in the present study. Manufacturer assay ID can be referred to as Thermo Fisher Scientific, USA.

|  | Gene Name | Gene symbol | Assay ID |
| --- | --- | --- | --- |
| Target Gene | Chemokine (C-C motif) ligand 2 | Ccl2 | Rn00580555_m1 |
|  | Tumor necrosis factor alpha induced protein 6 | Tnfaip6 | Rn01753871_m1 |
|  | S100 calcium-binding protein A4 | S100a4 | Rn01451938_m1 |
|  | TIMP metallopeptidase inhibitor 1 | Timp1 | Rn00587558_m1 |
|  | Uncoupling protein 2 | Ucp2 | Rn01754856_m1 |
|  | Uncoupling protein 3 | Ucp3 | Rn00565874_m1 |
|  | Aquaporin 7 | Aqp7 | Rn00569727_m1 |
|  | Solute carrier family 25, member 34 | Slc25a34 | Rn01749543_m1 |
|  | Perilipin 5 | Plin5 | Rn01527506_m1 |
| Housekeeping genes | Hypoxanthine Phosphoribosyltransferase 1 | Hprt1 | Rn01527840_m1 |
|  | Adaptor-related protein complex 3, delta 1 subunit | Ap3d1 | Rn01452619_m1 |

Table S3. The list of the DEGs of the soleus and EDL muscle

| Gene ID | Gene Symbol | Type | Fold-change log2 (CS/SA) | Q-value (CS/SA) |
| --- | --- | --- | --- | --- |
| DEGs of Soleus | | | | |
| 103693189 | 'LOC103693189' | mRNA | 22.845 | 1.25E-05 |
| 100909868 | 'LOC100909868' | mRNA | 22.354 | 2.32E-05 |
| 171577 | 'Epcam' | mRNA | 21.410 | 5.38E-05 |
| 114021 | 'Ebna1bp2' | mRNA | 21.358 | 5.41E-05 |
| 293669 | 'Cd248' | mRNA | 21.093 | 6.62E-05 |
| 24296 | 'Cyp1a1' | mRNA | 8.007 | 6.99E-05 |
| 100912573 | 'LOC100912573' | mRNA | 7.915 | 3.26E-05 |
| 100362027 | 'LOC100362027' | mRNA | 7.014 | 2.28E-03 |
| 102553270 | 'LOC102553270' | mRNA | 4.937 | 4.33E-02 |
| 287561 | 'Ccl7' | mRNA | 4.904 | 1.06E-02 |
| 308607 | 'E2f8' | mRNA | 4.704 | 5.36E-03 |
| 310344 | 'Plk4' | mRNA | 4.126 | 3.75E-02 |
| 362432 | 'Clec4d' | mRNA | 3.598 | 3.98E-02 |
| 282821 | 'Has1' | mRNA | 3.538 | 1.76E-02 |
| 24770 | 'Ccl2' | mRNA | 3.450 | 2.17E-02 |
| 25239 | 'Apod' | mRNA | 3.214 | 3.17E-02 |
| 114494 | 'Ccna2' | mRNA | 3.198 | 1.91E-02 |
| 58853 | 'Nr4a3' | mRNA | 3.153 | 1.64E-09 |
| 25453 | 'Gdnf' | mRNA | 3.082 | 1.41E-02 |
| 679837 | 'Adam12' | mRNA | 3.046 | 5.99E-03 |
| 686081 | 'B3galt2' | mRNA | 3.008 | 3.43E-02 |
| 50692 | 'Plaur' | mRNA | 2.993 | 8.96E-03 |
| 498638 | 'Msr1' | mRNA | 2.986 | 2.05E-02 |
| 295310 | 'Zfp697' | mRNA | 2.932 | 2.48E-03 |
| 83825 | 'Dclk1' | mRNA | 2.886 | 3.96E-03 |
| 24604 | 'Npy' | mRNA | 2.876 | 1.84E-02 |
| 690097 | 'LOC690097' | mRNA | 2.817 | 3.47E-02 |
| 499356 | 'Blnk' | mRNA | 2.804 | 1.59E-02 |
| 308761 | 'Prc1' | mRNA | 2.714 | 1.62E-04 |
| 24567 | 'Mt1' | mRNA | 2.703 | 5.38E-05 |
| 291234 | 'Mki67' | mRNA | 2.693 | 3.65E-02 |
| 362720 | 'Rrm2' | mRNA | 2.686 | 2.17E-02 |
| 689415 | 'Mt2A' | mRNA | 2.551 | 2.38E-04 |
| 306279 | 'Arhgap22' | mRNA | 2.532 | 6.87E-03 |
| 25665 | 'Scn5a' | mRNA | 2.521 | 4.33E-03 |
| 54237 | 'Cdk1' | mRNA | 2.503 | 2.56E-02 |
| 289211 | 'Fcgr2b' | mRNA | 2.491 | 4.92E-02 |
| 494206 | 'Ly49si1' | mRNA | 2.480 | 3.03E-03 |
| 498022 | 'RGD1559482' | mRNA | 2.406 | 1.46E-02 |
| 24914 | 'Lox' | mRNA | 2.374 | 3.79E-03 |
| 287813 | 'Igsf7' | mRNA | 2.357 | 3.53E-02 |
| 108348047 | 'LOC108348047' | mRNA | 2.354 | 1.06E-02 |
| 116510 | 'Timp1' | mRNA | 2.324 | 8.88E-04 |
| 361422 | 'Cotl1' | mRNA | 2.292 | 1.03E-02 |
| 84397 | 'Tnfaip6' | mRNA | 2.253 | 3.73E-02 |
| 310663 | 'Tnfaip8l2' | mRNA | 2.223 | 1.45E-02 |
| 310552 | 'Sfrp2' | mRNA | 2.209 | 1.73E-03 |
| 64045 | Glrx' | mRNA | 2.195 | 2.31E-02 |
| 89788 | 'Gna15' | mRNA | 2.171 | 4.37E-02 |
| 312897 | 'Msc' | mRNA | 2.088 | 2.58E-02 |
| 362430 | 'Clec4a1' | mRNA | 2.040 | 2.70E-02 |
| 29592 | 'Bcat1' | mRNA | 2.018 | 4.20E-02 |
| 291327 | 'Mrc1' | mRNA | 2.004 | 1.03E-02 |
| 365871 | 'Ciart' | mRNA | 1.991 | 1.86E-04 |
| 25441 | 'Fcer1g' | mRNA | 1.980 | 6.63E-03 |
| 291081 | 'Tubb2b' | mRNA | 1.942 | 3.37E-02 |
| 308586 | 'Sec1' | mRNA | 1.939 | 4.50E-03 |
| 293186 | 'Lyve1' | mRNA | 1.930 | 4.84E-02 |
| 289104 | 'Prg4' | mRNA | 1.921 | 3.74E-05 |
| 445442 | 'Thbs1' | mRNA | 1.886 | 1.41E-02 |
| 66026 | 'Trpv4' | mRNA | 1.881 | 1.36E-02 |
| 303378 | 'Slfn13' | mRNA | 1.881 | 1.20E-03 |
| 500059 | 'Prrt4' | mRNA | 1.880 | 4.66E-02 |
| 300870 | 'Fam46a' | mRNA | 1.820 | 3.13E-05 |
| 80850 | 'Gng2' | mRNA | 1.796 | 2.05E-02 |
| 359726 | 'Rnasel' | mRNA | 1.782 | 4.66E-02 |
| 364396 | 'Arl11' | mRNA | 1.777 | 1.39E-02 |
| 287435 | 'Cd68' | mRNA | 1.762 | 2.43E-02 |
| 309653 | 'Fgd2' | mRNA | 1.747 | 3.75E-02 |
| 114122 | 'Vcan' | mRNA | 1.746 | 1.37E-02 |
| 500040 | 'Tes' | mRNA | 1.741 | 1.70E-02 |
| 29639 | 'Fxyd2' | mRNA | 1.740 | 3.21E-02 |
| 100910688 | 'LOC100910688' | mRNA | 1.730 | 4.84E-02 |
| 360509 | 'Dock2' | mRNA | 1.699 | 3.78E-02 |
| 89783 | 'Laptm5' | mRNA | 1.683 | 1.06E-02 |
| 24587 | 'Nefh' | mRNA | 1.676 | 1.03E-02 |
| 60327 | 'F13a1' | mRNA | 1.663 | 2.21E-02 |
| 25155 | 'Syk' | mRNA | 1.625 | 2.78E-02 |
| 24546 | 'Slco2a1' | mRNA | 1.602 | 3.98E-02 |
| 113955 | 'Gpnmb' | mRNA | 1.600 | 3.66E-02 |
| 24615 | 'S100a4' | mRNA | 1.591 | 1.57E-07 |
| 360518 | 'Gfpt2' | mRNA | 1.577 | 4.89E-04 |
| 59329 | 'Sik1' | mRNA | 1.561 | 1.03E-02 |
| 296872 | 'Eda2r' | mRNA | 1.551 | 3.57E-02 |
| 309186 | 'Fermt3' | mRNA | 1.549 | 1.70E-02 |
| 361187 | 'Asb5' | mRNA | 1.546 | 1.24E-02 |
| 64195 | 'Clec10a' | mRNA | 1.537 | 1.37E-02 |
| 56611 | 'Anxa2' | mRNA | 1.536 | 1.91E-02 |
| 192203 | 'Fap' | mRNA | 1.510 | 4.66E-02 |
| 500906 | 'Cyth4' | mRNA | 1.479 | 3.68E-02 |
| 315714 | 'Loxl1' | mRNA | 1.469 | 8.96E-03 |
| 367313 | 'Col6a3' | mRNA | 1.467 | 1.58E-03 |
| 25439 | 'F2r' | mRNA | 1.464 | 3.66E-02 |
| 686809 | 'Exoc3l2' | mRNA | 1.455 | 1.83E-02 |
| 83727 | 'Fbn1' | mRNA | 1.451 | 4.89E-04 |
| 85246 | 'Gas7' | mRNA | 1.442 | 5.99E-03 |
| 81505 | 'Emp3' | mRNA | 1.435 | 2.69E-02 |
| 58949 | 'Ptafr' | mRNA | 1.411 | 2.19E-03 |
| 366960 | 'Maff' | mRNA | 1.407 | 1.24E-02 |
| 25648 | 'Slc7a1' | mRNA | 1.389 | 1.68E-02 |
| 58919 | 'Ccnd1' | mRNA | 1.388 | 6.87E-03 |
| 302642 | 'Sat1' | mRNA | 1.385 | 7.67E-03 |
| 84410 | 'Klf5' | mRNA | 1.379 | 1.62E-04 |
| 64158 | 'Tuba1a' | mRNA | 1.359 | 1.01E-03 |
| 311437 | 'Rassf2' | mRNA | 1.339 | 1.06E-02 |
| 81826 | 'Slc20a1' | mRNA | 1.337 | 6.83E-06 |
| 303501 | 'Arhgap23' | mRNA | 1.317 | 3.47E-02 |
| 502776 | 'Scrn1' | mRNA | 1.315 | 1.10E-05 |
| 25181 | 'Bgn' | mRNA | 1.296 | 3.20E-03 |
| 25314 | 'Emp1' | mRNA | 1.292 | 1.46E-02 |
| 245956 | 'Scn3b' | mRNA | 1.273 | 4.90E-02 |
| 81778 | 'S100a10' | mRNA | 1.269 | 9.01E-03 |
| 84032 | 'Col3a1' | mRNA | 1.263 | 7.61E-03 |
| 100363145 | 'Stab1' | mRNA | 1.259 | 4.24E-04 |
| 301416 | 'Coq10b' | mRNA | 1.228 | 2.76E-02 |
| 79128 | 'Dab2' | mRNA | 1.220 | 1.84E-02 |
| 54315 | 'Ucp2' | mRNA | 1.213 | 3.74E-02 |
| 103692066 | 'LOC103692066' | mRNA | 1.142 | 2.04E-02 |
| 60338 | 'Fxyd5' | mRNA | 1.141 | 2.44E-02 |
| 116487 | 'Tgfbi' | mRNA | 1.128 | 4.89E-04 |
| 114208 | 'Skil' | mRNA | 1.118 | 1.01E-03 |
| 25267 | 'Pdgfra' | mRNA | 1.094 | 3.06E-02 |
| 24915 | 'Pdlim4' | mRNA | 1.093 | 1.06E-02 |
| 85490 | 'Col5a1' | mRNA | 1.088 | 8.34E-03 |
| 690899 | 'Vsir' | mRNA | 1.079 | 1.06E-02 |
| 361680 | 'Lsp1' | mRNA | 1.074 | 1.06E-02 |
| 406864 | 'Clic1' | mRNA | 1.071 | 2.94E-02 |
| 500929 | 'Tuba1b' | mRNA | 1.070 | 1.46E-02 |
| 683788 | 'Fscn1' | mRNA | 1.049 | 2.17E-02 |
| 83792 | 'Scd2' | mRNA | 1.047 | 1.46E-02 |
| 29376 | 'Irs2' | mRNA | 1.032 | 7.61E-03 |
| 114519 | 'Nfil3' | mRNA | 1.029 | 3.79E-03 |
| 81660 | 'Gatm' | mRNA | 1.026 | 3.23E-02 |
| 65161 | 'Litaf' | mRNA | 1.015 | 2.05E-02 |
| 246775 | 'Tnfsf10' | mRNA | -1.003 | 3.78E-02 |
| 64526 | 'Ech1' | mRNA | -1.010 | 3.96E-03 |
| 29740 | 'Eci1' | mRNA | -1.012 | 3.39E-02 |
| 282636 | 'Impa2' | mRNA | -1.015 | 1.36E-02 |
| 29653 | 'Gpam' | mRNA | -1.049 | 7.34E-03 |
| 360634 | 'Plekhh3' | mRNA | -1.054 | 1.24E-02 |
| 54231 | 'Car2' | mRNA | -1.058 | 9.65E-03 |
| 108348111 | 'LOC108348111' | mRNA | -1.108 | 1.59E-02 |
| 298606 | 'Slc25a34' | mRNA | -1.180 | 5.15E-04 |
| 100362676 | 'Klhl33' | mRNA | -1.184 | 3.68E-02 |
| 363331 | 'Plin4' | mRNA | -1.191 | 7.10E-03 |
| 100912034 | 'LOC100912034' | mRNA | -1.218 | 4.50E-03 |
| 29577 | 'Hes1' | mRNA | -1.245 | 1.53E-03 |
| 100363484 | 'Ddit4l' | mRNA | -1.247 | 4.84E-02 |
| 313558 | 'Foxo6' | mRNA | -1.290 | 4.58E-02 |
| 85239 | 'Mlycd' | mRNA | -1.290 | 4.05E-02 |
| 114244 | 'Hcn2' | mRNA | -1.306 | 1.20E-02 |
| 29171 | 'Aqp7' | mRNA | -1.306 | 1.10E-05 |
| 501283 | 'Plin5' | mRNA | -1.344 | 3.97E-02 |
| 684480 | 'Btnl9' | mRNA | -1.350 | 8.80E-03 |
| 246298 | 'Retsat' | mRNA | -1.414 | 1.10E-05 |
| 361824 | 'Chchd10' | mRNA | -1.439 | 5.54E-03 |
| 24953 | 'Gcgr' | mRNA | -1.493 | 4.20E-02 |
| 289491 | 'Mrpl1' | mRNA | -1.493 | 3.98E-02 |
| 313917 | 'Abhd1' | mRNA | -1.758 | 1.06E-02 |
| 500750 | 'Tmem196' | mRNA | -1.814 | 2.31E-02 |
| 171085 | 'Pcsk4' | mRNA | -1.846 | 3.47E-02 |
| 289388 | 'G0s2' | mRNA | -2.223 | 1.00E-03 |
| 25708 | 'Ucp3' | mRNA | -2.330 | 6.51E-05 |
| 100910978 | 'LOC100910978' | mRNA | -4.951 | 1.20E-02 |
| 689064 | 'LOC689064' | mRNA | -21.605 | 4.77E-05 |
| DEGs of EDL | | | | |
| 100362027 | 'LOC100362027' | mRNA | 7.318626275 | 0.005987691 |
| 24296 | 'Cyp1a1' | mRNA | 6.53416612 | 3.05E-06 |
| 497979 | 'Tmem100' | mRNA | 1.957742084 | 1.04E-07 |
| 690195 | 'Prima1' | mRNA | 1.873680468 | 9.35E-06 |
| 24626 | 'Pde4b' | mRNA | 1.716048184 | 3.95E-04 |
| 252917 | 'Nr1d1' | mRNA | 1.350638189 | 0.005987691 |
| 84489 | 'Fgfr3' | mRNA | 1.31567244 | 0.005987691 |
| 362196 | 'Chac1' | mRNA | 1.079508609 | 0.03642997 |
| 304266 | 'Gbp1' | mRNA | -2.04620795 | 1.35E-04 |
| 293862 | 'Fam50a' | mRNA | -21.36350477 | 1.55E-04 |

| Hug Gene | Degree | Closeness | Betweenness | Stress | Clustering Coefficient | log fold-change | Q-value |
| --- | --- | --- | --- | --- | --- | --- | --- |
| Ccl2 | 21 | 51.0 | 3435.0 | 8772.0 | 0.21 | 3.5 | 0.022 |
| Timp1 | 18 | 47.1 | 899.2 | 3352.0 | 0.33 | 2.3 | 0.001 |
| Cd68 | 17 | 46.1 | 834.5 | 3582.0 | 0.33 | 1.8 | 0.024 |
| Pdgfra | 15 | 45.1 | 812.0 | 2990.0 | 0.31 | 1.1 | 0.031 |
| Lox | 15 | 44.7 | 789.5 | 3208.0 | 0.40 | 2.4 | 0.004 |
| Col3a1 | 13 | 42.7 | 232.3 | 1458.0 | 0.56 | 1.3 | 0.008 |
| Ccnd1 | 12 | 43.1 | 1891.2 | 5894.0 | 0.15 | 1.4 | 0.007 |
| Thbs1 | 12 | 41.6 | 470.8 | 1850.0 | 0.42 | 1.9 | 0.014 |
| Bgn | 12 | 39.0 | 254.9 | 1180.0 | 0.48 | 1.3 | 0.003 |
| Fcer1g | 11 | 39.4 | 959.6 | 2958.0 | 0.16 | 2.0 | 0.007 |

Table S4. Top10 hub genes identified in DEGs of soleus

Table S5. List of the significantly enriched GSEA results of the soleus and EDL muscle

| ID | Description | | setSize | NES | FDR |  |  |
| --- | --- | --- | --- | --- | --- | --- | --- |
| Pathway - Soleus (up-regulated) | | | | | | | |
| 04520 | Adherens junction | | 70 | 2.091 | <0.001 | 4724 | tags=59%, list=27%, signal=80% |
| 04380 | Osteoclast differentiation | | 117 | 2.006 | <0.001 | 4202 | tags=50%, list=24%, signal=66% |
| 04666 | Fc gamma R-mediated phagocytosis | | 94 | 1.966 | 0.001 | 3421 | tags=50%, list=19%, signal=62% |
| 04650 | Natural killer cell mediated cytotoxicity | | 85 | 1.949 | 0.001 | 3133 | tags=46%, list=18%, signal=56% |
| 04662 | B cell receptor signaling pathway | | 75 | 1.906 | 0.001 | 3154 | tags=43%, list=18%, signal=52% |
| 04115 | p53 signaling pathway | | 72 | 1.909 | 0.001 | 4150 | tags=53%, list=24%, signal=69% |
| 04064 | NF-kappa B signaling pathway | | 93 | 1.898 | 0.002 | 4108 | tags=49%, list=23%, signal=64% |
| 04625 | C-type lectin receptor signaling pathway | | 100 | 1.871 | 0.002 | 4322 | tags=44%, list=25%, signal=58% |
| 04611 | Platelet activation | | 122 | 1.863 | 0.002 | 3668 | tags=40%, list=21%, signal=50% |
| 04062 | Chemokine signaling pathway | | 164 | 1.841 | 0.002 | 4500 | tags=48%, list=26%, signal=63% |
| 04110 | Cell cycle | | 125 | 1.857 | 0.002 | 5943 | tags=59%, list=34%, signal=89% |
| 04145 | Phagosome | | 158 | 1.820 | 0.002 | 3718 | tags=44%, list=21%, signal=55% |
| 04810 | Regulation of actin cytoskeleton | | 205 | 1.809 | 0.002 | 5071 | tags=52%, list=29%, signal=72% |
| 04540 | Gap junction | | 81 | 1.806 | 0.002 | 3838 | tags=41%, list=22%, signal=52% |
| 04670 | Leukocyte transendothelial migration | | 106 | 1.799 | 0.003 | 4542 | tags=51%, list=26%, signal=68% |
| 04664 | Fc epsilon RI signaling pathway | | 60 | 1.787 | 0.003 | 3446 | tags=43%, list=20%, signal=54% |
| 04215 | Apoptosis - multiple species | | 32 | 1.775 | 0.003 | 3594 | tags=50%, list=20%, signal=63% |
| 04978 | Mineral absorption | | 51 | 1.771 | 0.003 | 4147 | tags=49%, list=24%, signal=64% |
| 04060 | Cytokine-cytokine receptor interaction | | 205 | 1.771 | 0.003 | 4055 | tags=40%, list=23%, signal=51% |
| 04660 | T cell receptor signaling pathway | | 94 | 1.756 | 0.004 | 4503 | tags=46%, list=26%, signal=61% |
| 04640 | Hematopoietic cell lineage | | 84 | 1.745 | 0.005 | 3116 | tags=39%, list=18%, signal=48% |
| 04510 | Focal adhesion | | 195 | 1.739 | 0.005 | 5346 | tags=54%, list=30%, signal=77% |
| 04610 | Complement and coagulation cascades | | 65 | 1.699 | 0.008 | 5069 | tags=52%, list=29%, signal=73% |
| 04151 | PI3K-Akt signaling pathway | | 313 | 1.690 | 0.009 | 4246 | tags=40%, list=24%, signal=52% |
| 04071 | Sphingolipid signaling pathway | | 117 | 1.677 | 0.010 | 3821 | tags=36%, list=22%, signal=46% |
| 04061 | Viral protein interaction with cytokine and cytokine receptor | | 69 | 1.664 | 0.011 | 3910 | tags=41%, list=22%, signal=52% |
| 04141 | Protein processing in endoplasmic reticulum | | 164 | 1.657 | 0.012 | 6169 | tags=56%, list=35%, signal=86% |
| 04668 | TNF signaling pathway | | 106 | 1.659 | 0.012 | 5876 | tags=61%, list=33%, signal=92% |
| 00601 | Glycosphingolipid biosynthesis - lacto and neolacto series | | 25 | 1.642 | 0.014 | 3867 | tags=52%, list=22%, signal=67% |
| 04672 | Intestinal immune network for IgA production | | 34 | 1.639 | 0.014 | 2402 | tags=32%, list=14%, signal=37% |
| 04210 | Apoptosis | | 131 | 1.630 | 0.015 | 3683 | tags=40%, list=21%, signal=50% |
| 04142 | Lysosome | | 126 | 1.626 | 0.015 | 5793 | tags=44%, list=33%, signal=66% |
| 04015 | Rap1 signaling pathway | | 198 | 1.621 | 0.016 | 2972 | tags=33%, list=17%, signal=39% |
| 04361 | Axon regeneration | | 89 | 1.604 | 0.018 | 4840 | tags=39%, list=28%, signal=54% |
| 04621 | NOD-like receptor signaling pathway | | 151 | 1.605 | 0.018 | 4320 | tags=40%, list=25%, signal=52% |
| 04926 | Relaxin signaling pathway | | 116 | 1.601 | 0.018 | 4482 | tags=42%, list=26%, signal=56% |
| 04657 | IL-17 signaling pathway | | 76 | 1.586 | 0.021 | 6278 | tags=58%, list=36%, signal=90% |
| 04010 | MAPK signaling pathway | | 279 | 1.581 | 0.022 | 4500 | tags=42%, list=26%, signal=55% |
| 00100 | Steroid biosynthesis | | 19 | 1.582 | 0.022 | 5182 | tags=68%, list=30%, signal=97% |
| 04214 | Apoptosis - fly | | 50 | 1.579 | 0.022 | 5595 | tags=58%, list=32%, signal=85% |
| 04659 | Th17 cell differentiation | | 95 | 1.572 | 0.024 | 4213 | tags=39%, list=24%, signal=51% |
| 04014 | Ras signaling pathway | | 214 | 1.568 | 0.025 | 4500 | tags=42%, list=26%, signal=56% |
| 04620 | Toll-like receptor signaling pathway | | 85 | 1.564 | 0.026 | 3154 | tags=34%, list=18%, signal=41% |
| 04370 | VEGF signaling pathway | | 56 | 1.555 | 0.027 | 4202 | tags=43%, list=24%, signal=56% |
| 04630 | JAK-STAT signaling pathway | | 125 | 1.544 | 0.030 | 4057 | tags=40%, list=23%, signal=52% |
| 04914 | Progesterone-mediated oocyte maturation | | 87 | 1.541 | 0.030 | 4605 | tags=39%, list=26%, signal=53% |
| 04512 | ECM-receptor interaction | | 85 | 1.527 | 0.032 | 5778 | tags=55%, list=33%, signal=82% |
| 04392 | Hippo signaling pathway - multiple species | | 27 | 1.529 | 0.033 | 4697 | tags=59%, list=27%, signal=81% |
| 04613 | Neutrophil extracellular trap formation | | 150 | 1.531 | 0.033 | 3082 | tags=31%, list=18%, signal=37% |
| 04725 | Cholinergic synapse | | 101 | 1.519 | 0.034 | 4202 | tags=39%, list=24%, signal=50% |
| 04114 | Oocyte meiosis | | 110 | 1.516 | 0.035 | 5193 | tags=41%, list=30%, signal=58% |
| 04072 | Phospholipase D signaling pathway | | 139 | 1.511 | 0.035 | 4492 | tags=37%, list=26%, signal=50% |
| 04013 | MAPK signaling pathway - fly | | 82 | 1.512 | 0.035 | 4695 | tags=45%, list=27%, signal=61% |
| 04612 | Antigen processing and presentation | | 70 | 1.513 | 0.035 | 4290 | tags=47%, list=24%, signal=62% |
| 04918 | Thyroid hormone synthesis | | 65 | 1.509 | 0.035 | 3685 | tags=38%, list=21%, signal=48% |
| 04068 | FoxO signaling pathway | | 126 | 1.498 | 0.038 | 4300 | tags=40%, list=24%, signal=52% |
| 04012 | ErbB signaling pathway | | 82 | 1.494 | 0.039 | 4482 | tags=39%, list=26%, signal=52% |
| 04911 | Insulin secretion | | 73 | 1.486 | 0.042 | 3733 | tags=34%, list=21%, signal=43% |
| 04915 | Estrogen signaling pathway | | 113 | 1.478 | 0.044 | 4396 | tags=39%, list=25%, signal=52% |
| 04350 | TGF-beta signaling pathway | | 90 | 1.462 | 0.049 | 4530 | tags=40%, list=26%, signal=54% |
| 04728 | Dopaminergic synapse | | 122 | 1.464 | 0.049 | 5638 | tags=43%, list=32%, signal=62% |
| 04727 | GABAergic synapse | | 80 | 1.462 | 0.049 | 4046 | tags=39%, list=23%, signal=50% |
| 04514 | Cell adhesion molecules | | 145 | 1.459 | 0.050 | 5869 | tags=52%, list=33%, signal=78% |
| 04929 | GnRH secretion | | 59 | 1.459 | 0.050 | 4202 | tags=39%, list=24%, signal=51% |
|  |  | |  |  |  |  |  |
| Pathway - Soleus (down-regulated) | | | | | | | |
| 190 | Oxidative phosphorylation | | 120 | -2.940 | <0.001 | 3000 | tags=57%, list=17%, signal=68% |
| 4146 | Peroxisome | | 80 | -2.798 | <0.001 | 2211 | tags=50%, list=13%, signal=57% |
| 20 | Citrate cycle (TCA cycle) | | 29 | -2.728 | <0.001 | 2167 | tags=62%, list=12%, signal=71% |
| 280 | Valine, leucine and isoleucine degradation | | 52 | -2.657 | <0.001 | 2596 | tags=58%, list=15%, signal=67% |
| 640 | Propanoate metabolism | | 30 | -2.546 | <0.001 | 2419 | tags=63%, list=14%, signal=73% |
| 3320 | PPAR signaling pathway | | 72 | -2.478 | <0.001 | 2583 | tags=51%, list=15%, signal=60% |
| 71 | Fatty acid degradation | | 44 | -2.424 | <0.001 | 2446 | tags=52%, list=14%, signal=61% |
| 630 | Glyoxylate and dicarboxylate metabolism | | 28 | -2.362 | <0.001 | 3078 | tags=71%, list=18%, signal=86% |
| 650 | Butanoate metabolism | | 25 | -2.231 | <0.001 | 2092 | tags=44%, list=12%, signal=50% |
| 1200 | Carbon metabolism | | 107 | -2.230 | <0.001 | 3078 | tags=45%, list=18%, signal=54% |
| 620 | Pyruvate metabolism | | 40 | -2.225 | <0.001 | 2889 | tags=48%, list=16%, signal=57% |
| 4714 | Thermogenesis | | 212 | -2.220 | <0.001 | 3626 | tags=51%, list=21%, signal=64% |
| 120 | Primary bile acid biosynthesis | | 15 | -1.984 | 0.003 | 2051 | tags=53%, list=12%, signal=60% |
| 970 | Aminoacyl-tRNA biosynthesis | | 45 | -1.923 | 0.003 | 4294 | tags=56%, list=24%, signal=73% |
| 1212 | Fatty acid metabolism | | 60 | -1.935 | 0.003 | 2588 | tags=40%, list=15%, signal=47% |
| 982 | Drug metabolism - cytochrome P450 | | 47 | -1.897 | 0.004 | 2446 | tags=36%, list=14%, signal=42% |
| 1210 | 2-Oxocarboxylic acid metabolism | | 17 | -1.831 | 0.007 | 2798 | tags=71%, list=16%, signal=84% |
| 270 | Cysteine and methionine metabolism | | 46 | -1.765 | 0.009 | 2112 | tags=33%, list=12%, signal=37% |
| 450 | Selenocompound metabolism | | 17 | -1.773 | 0.009 | 3763 | tags=59%, list=21%, signal=75% |
| 983 | Drug metabolism - other enzymes | | 70 | -1.662 | 0.020 | 3512 | tags=40%, list=20%, signal=50% |
| 1230 | Biosynthesis of amino acids | | 71 | -1.601 | 0.027 | 2839 | tags=37%, list=16%, signal=44% |
| 380 | Tryptophan metabolism | | 43 | -1.607 | 0.028 | 2167 | tags=40%, list=12%, signal=45% |
| 260 | Glycine, serine and threonine metabolism | | 36 | -1.590 | 0.029 | 3089 | tags=44%, list=18%, signal=54% |
| 730 | Thiamine metabolism | | 15 | -1.580 | 0.029 | 3165 | tags=40%, list=18%, signal=49% |
| 62 | Fatty acid elongation | | 29 | -1.535 | 0.036 | 542 | tags=21%, list=3%, signal=21% |
| 980 | Metabolism of xenobiotics by cytochrome P450 | | 48 | -1.537 | 0.037 | 2446 | tags=29%, list=14%, signal=34% |
| 4260 | Cardiac muscle contraction | | 78 | -1.511 | 0.041 | 2969 | tags=38%, list=17%, signal=46% |
| 4723 | Retrograde endocannabinoid signaling | | 128 | -1.479 | 0.043 | 3356 | tags=41%, list=19%, signal=51% |
| 61 | Fatty acid biosynthesis | | 18 | -1.484 | 0.044 | 3198 | tags=50%, list=18%, signal=61% |
| 410 | beta-Alanine metabolism | | 28 | -1.487 | 0.045 | 4262 | tags=57%, list=24%, signal=75% |
| 770 | Pantothenate and CoA biosynthesis | | 20 | -1.484 | 0.045 | 921 | tags=30%, list=5%, signal=32% |
|  |  | |  |  |  |  |  |
| Pathway -Extensor digitorum longus (up-regulated) | | | | | | | |
| 563 | Glycosylphosphatidylinositol (GPI)-anchor biosynthesis | | 26 | 2.025 | 0.008 | 4252 | tags=58%, list=26%, signal=77% |
| 260 | Glycine, serine and threonine metabolism | | 34 | 1.828 | 0.041 | 939 | tags=24%, list=6%, signal=25% |
|  |  |  | | | | | |
| Pathway -Extensor digitorum longus (down-regulated) | | | | | | | |
| 4612 | Antigen processing and presentation | | 65 | -1.905 | 0.005 | 2450 | tags=38%, list=15%, signal=45% |
| 4672 | Intestinal immune network for IgA production | | 31 | -1.862 | 0.008 | 1878 | tags=39%, list=11%, signal=44% |
| 240 | Pyrimidine metabolism | | 49 | -1.819 | 0.010 | 4178 | tags=47%, list=25%, signal=62% |
| 120 | Primary bile acid biosynthesis | | 15 | -1.829 | 0.010 | 2413 | tags=60%, list=14%, signal=70% |
| 4146 | Peroxisome | | 77 | -1.776 | 0.016 | 2519 | tags=38%, list=15%, signal=44% |
| 71 | Fatty acid degradation | | 41 | -1.723 | 0.026 | 2869 | tags=44%, list=17%, signal=53% |
| 4640 | Hematopoietic cell lineage | | 74 | -1.665 | 0.037 | 1176 | tags=20%, list=7%, signal=22% |

Note: NES: normalized enrichment score. FDR: false discovery rate. NES with positive value: up-regulated in the treatment group (CS group); NES with negative value: down-regulated in the treatment group.


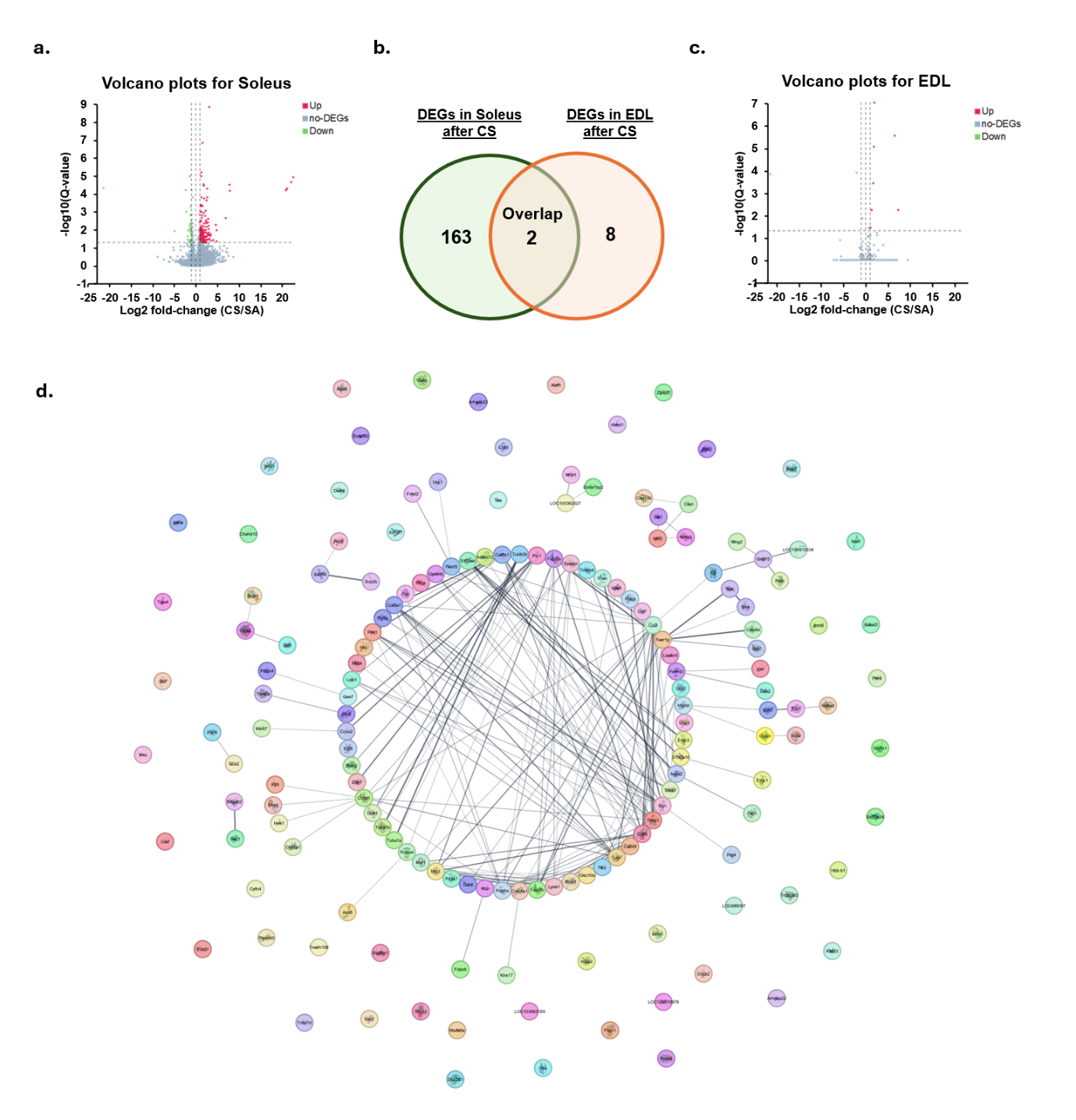


Figure. S1 Volcano plots and Venn diagram of significantly expressed genes in the soleus and Extensor Digitorum Longus (EDL)

(a) Volcano plots significantly expressed genes in the soleus (b) Venn diagram of gene intersection between the soleus and EDL (c) Volcano plots significantly expressed genes in the EDL. The color of the dots in volcano plots indicates a gene with no significant difference (gray), that has been up-regulated (red), and that has been down-regulated (green).


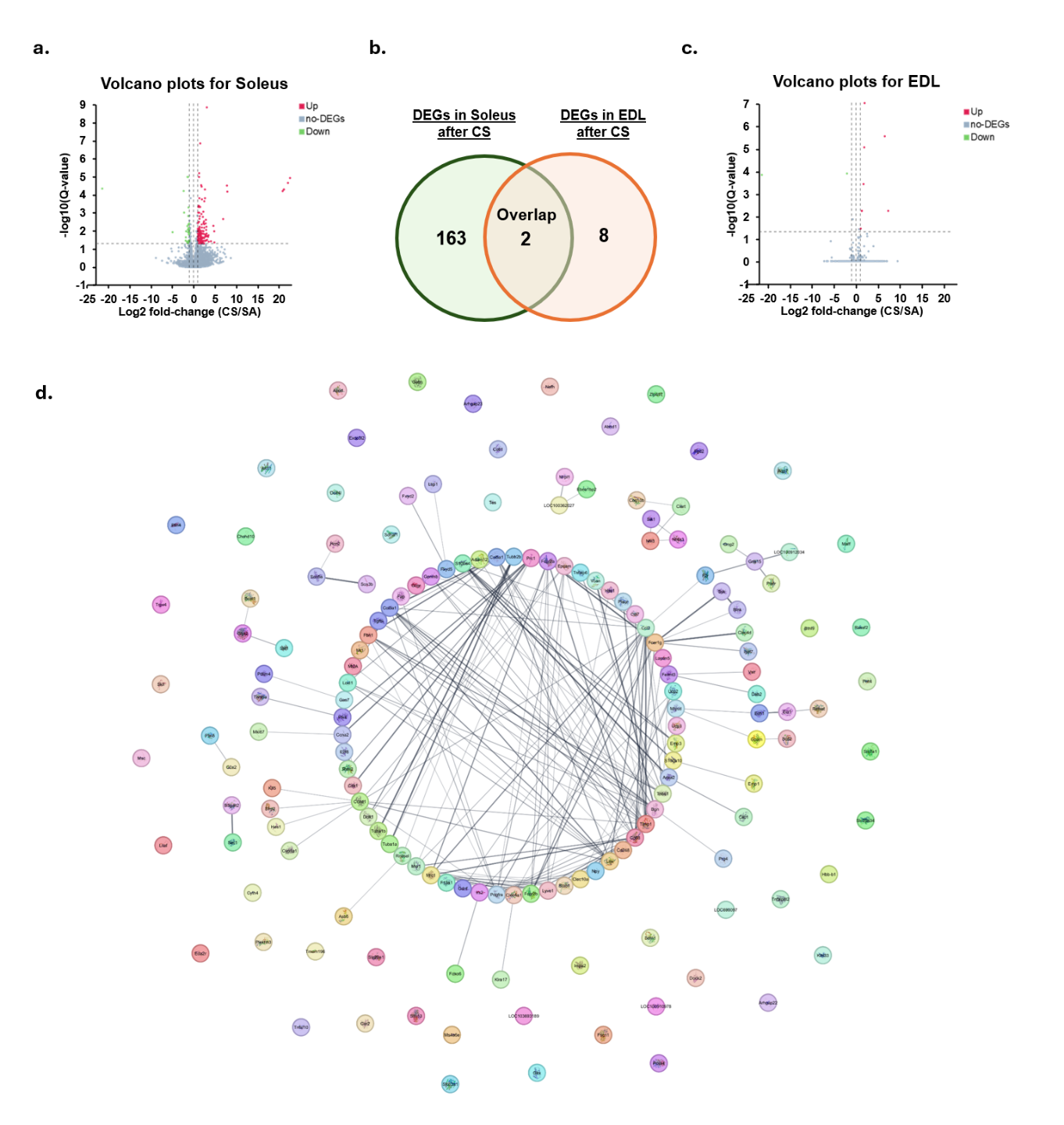


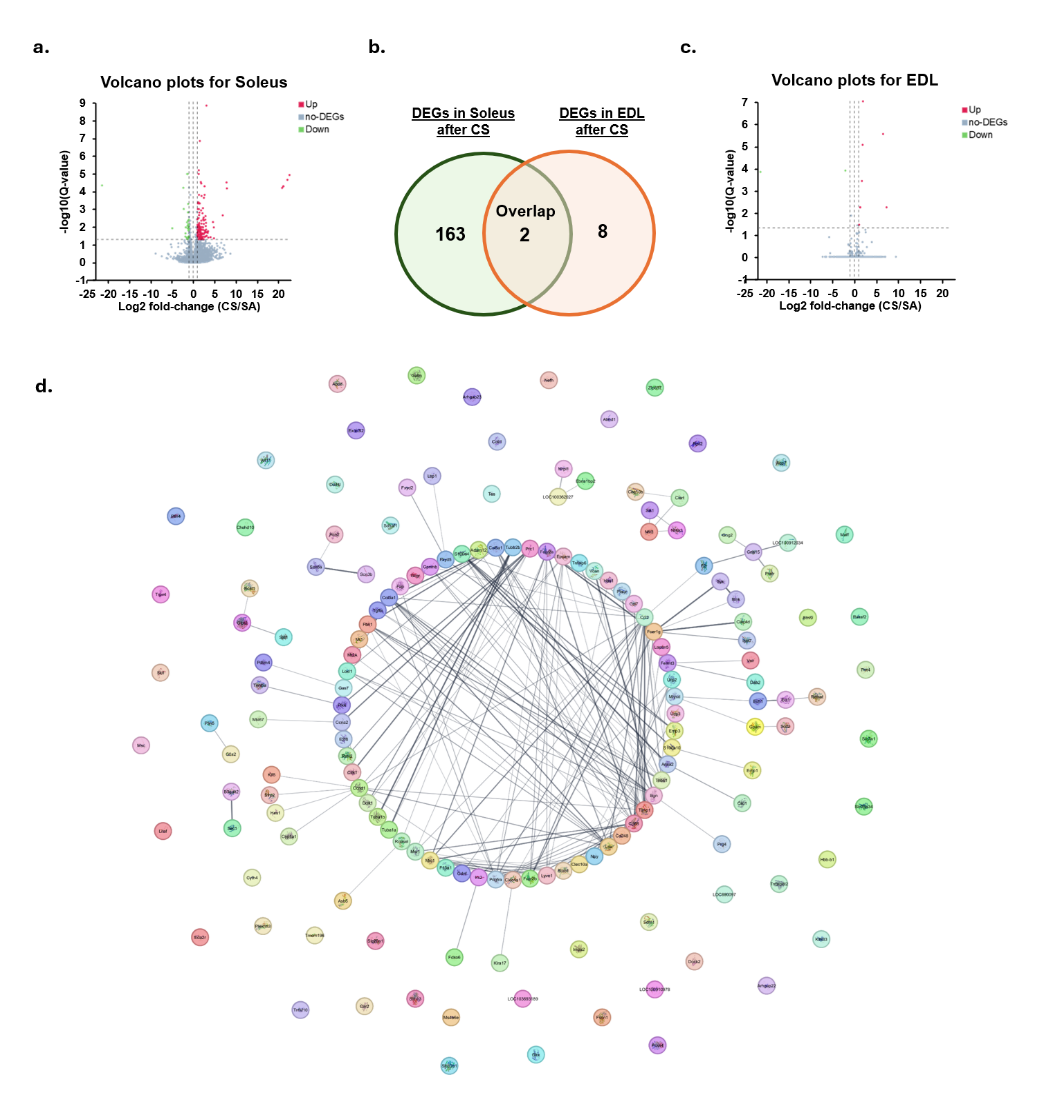


Figure. S2 PPI networks of DEGs in the soleus.

(a) A total of 165 DEGs were imported into the STRING online database and the PPI network was obtained with 234 edges.
